# Linking Functional Traits To Trophic Roles In Scavenger Assemblages

**DOI:** 10.1002/ece3.70485

**Published:** 2025-01-08

**Authors:** Violeta Marie Montenegro, Patricia Mateo‐Tomás, Jessica Schneider, Daisy H. Dent, Tom Crowther, Carolina Bello

**Affiliations:** ^1^ Department of Environmental Systems Science ETH Zürich Switzerland; ^2^ Biodiversity Research Institute (University of Oviedo – CSIC – Principado de Asturias) Mieres Spain; ^3^ Smithsonian Tropical Research Institute Panama Panama; ^4^ Max Planck Institute for Animal Behavior Konstanz Germany

**Keywords:** carcasses, functional traits, interaction networks, scavengers, Spain, trophic role

## Abstract

Scavenging is a widespread feeding strategy involving a diversity of taxa from different trophic levels, from apex predators to obligate scavengers. Scavenger species play a crucial role in ecosystem functioning by removing carcasses, recycling nutrients and preventing disease spread. Understanding the trophic roles of scavenger species can help identify specialized species with unique roles and species that may be more vulnerable to ecological changes. To identify species with specialized roles, we studied three scavenger networks (one in north temperate Spain and two in central‐south Mediterranean Spain) that comprised 25 scavenger species (65% birds and 35% mammals), consuming carcasses of four wild ungulate species. We characterized the trophic role of a species by combining four species‐level network metrics (normalized degree, specialization, closeness, and betweenness centrality) into a single centrality metric, quantifying how scavenger species interact with carcass species within their ecological network. Higher centrality indicates the species feeds on a greater variety of carcasses and may contribute more to carrion consumption than species with lower centrality, which have more peripheral and specialized roles. The griffon vulture (*Gyps fulvus*) and the azure‐winged magpie (*Cyanopica cyanus*) had the highest centrality. In contrast, the red kite (*Milvus milvus*) in the northern site had the lowest centrality, and the Egyptian vulture (*Neophron percnopterus*) was among the most peripheral species for all three networks. In general, scavengers with large home ranges and nocturnal or crepuscular activity patterns tended to have more central roles, whereas species that forage silently tended to have more peripheral roles. Changes in species' centrality between sites and the high centrality of species with large home ranges suggest that management strategies in one location can have implications that extend beyond, highlighting the need to implement coordinated transboundary protection efforts to ensure the resilience and functionality of scavenger networks and derived ecosystem services.

## Abstract 2

We did not plan for a graphical table of content.

## 1 Introduction

The consumption of dead animal matter (scavenging) is widespread among vertebrates and facilitates the flow of energy and nutrients across trophic levels (Wilson and Wolkovich 2011; Inger et al. 2016). By consuming dead animals, scavengers support essential ecosystem functions such as nutrient cycling and may prevent the spread of diseases associated with decaying matter (DeVault, Rhodes Olin, and Shivik 2003; O'Bryan et al. 2018). This scavenging role could contribute to reducing the economic and environmental costs of carcass transport and disposal, and of maintaining public health (Markandya et al. 2008; Morales‐Reyes et al. 2015; DeVault et al. 2016). Yet not all carcass‐consuming species contribute equally to these ecosystem functions (Mateo‐Tomás et al. 2017). Species contributions vary based on life‐history traits that drive broad versus specialized feeding, e.g., large home ranges or social foraging strategies, but also on species' abundance and distribution (Mateo‐Tomás et al. 2017; Gutiérrez‐Cánovas et al. 2020; Sebastián‐González et al. 2021).

The diverse feeding preferences of scavengers shape a complex network of interactions and promote resource partitioning, coexistence, and diversity (Mateo‐Tomás et al. 2017; Pardo‐Barquín, Mateo‐Tomás, and Olea 2019; Olea, Iglesias, and Mateo‐Tomás 2022). In general, the networks formed by species feeding on carrion exhibit a nested structure (Selva and Fortuna 2007; Sebastián‐González et al. 2020), meaning that the diet of specialist species is a subset of the diet of generalist species (Selva and Fortuna 2007). Functionally dominant scavengers able to rapidly consume most carcasses across ecosystems include abundant and widespread species (e.g., wild boar) as well as obligate scavengers like vultures, able to rapidly gather at carcasses (Mateo‐Tomás et al. 2017). Moreover, the extensive dispersal abilities of large vultures suggest that vertebrate scavenger communities across different ecosystems can be viewed as connected metacommunities (Mateo‐Tomás, Olea, López‐Bao, et al. 2019; Mateo‐Tomás, Olea, Selva, et al. 2019). Consequently, nestedness is observed not only within local communities but also at larger spatial scales (Selva and Fortuna 2007; Sebastián‐González et al. 2016; Mateo‐Tomás, Olea, López‐Bao, et al. 2019; Mateo‐Tomás, Olea, Selva, et al. 2019).

Nested structure was shown to increase the efficiency of carrion consumption and minimize competition among scavengers, allowing species with distinct roles to coexist on a carrion resource, especially in species‐rich assemblages (Selva and Fortuna 2007; Sebastián‐González et al. 2016). Hence, understanding community structure through network analyses can give us critical insights into the mechanisms driving ecosystem functioning and stability (Hooper et al. 2005; Namba, Tanabe, and Maeda 2008). In this network of interactions, a species' trophic role can be determined by its position in the network structure, which is quantified using centrality indices (Cirtwill et al. 2018). These indices measure how connected a species is within the network, thus allowing us to say that a species is central when it is more connected or peripheral when it is less connected. Thus, in scavenger networks, central species would be generalists that feed on a broad range of carcass species, whereas peripheral species are more specialized and engage with specific carcass types (Jordán, Liu, and Davis 2006). Central species' ability to access most carcasses may underscore an important role in maintaining network's functions such as nutrient recycling (Bascompte 2003). Moreover, central species maintain the network cohesive, acting as a bridge between other species (Cirtwill et al. 2018). In contrast, less connected species (peripheral) may provide unique functions crucial for maintaining ecosystem functioning that other species cannot replace (Dehling et al. 2021). Therefore, the species' position in the network represents varying levels of adaptation for locating and consuming carrion, characteristics strongly influenced by the functional traits inherent to each species (DeVault, Rhodes Olin, and Shivik 2003; Gutiérrez‐Cánovas et al. 2020).

Functional traits provide a valuable framework for understanding community structure and identifying common patterns across different ecosystems (McGill et al. 2006). By constraining the potential trophic interactions of a species within a network (Morales‐Castilla et al. 2015; Laigle et al. 2018), they provide mechanistic insights into the functional impacts of those species (Aubin et al. 2013; Bartomeus et al. 2016; Laigle et al. 2018). Consequently, functional traits are valuable for identifying species crucial to network structure and function (Coux et al. 2016; Moulatlet, Dáttilo, and Villalobos 2023). For instance, vultures exemplify unique traits tailored to scavenging, such as soaring flight, acute eyesight, and remarkably low stomach pH levels, which allow them to exclusively subsist on carrion as their primary food source (Ruxton and Houston 2004; Grigg et al. 2017; Zepeda Mendoza et al. 2018). These traits allow vultures to interact with a wide range of carcasses, making them generalists within the carrion removal process and positioning them more centrally within scavenger networks compared to most facultative scavengers. As a result, vultures are likely to have a disproportionately important role in the functioning of scavenger networks, with varying impacts across different environments. As such, vultures may outcompete other scavengers under certain conditions (e.g., at supplementary feeding places or in open landscapes; Cortés‐Avizanda et al. 2016; Mateo‐Tomás et al. 2017; Pardo‐Barquín, Mateo‐Tomás, and Olea 2019), but they may also facilitate the scavenging activity of other species by opening thick‐skinned carcasses and making them accessible to smaller scavengers (Moleón et al. 2014), thereby supporting the diversity of scavengers at carrion. Moreover, other traits, such as range size and local abundance, are also linked to species' centrality in ecological networks. For instance, Moulatlet, Dáttilo, and Villalobos (2023) found that a large range size increases a bird species' interactions with a broader set of plants across its distribution, thus enhancing its centrality. Similarly, Laurindo et al. (2020) identified abundance as a primary determinant of a species' ecological role within seed‐dispersal networks. However, despite the growing understanding of the traits that enable the process of scavenging in various species (e.g., large home ranges, soaring flight; Ruxton and Houston 2004, Gutiérrez‐Cánovas et al. 2020), it is still not clear how these are associated with central, unique, or peripheral roles across the network, and if the role of a given scavenger and determining traits, change between locations.

Here, we used empirical data from scavenger networks at three sites in temperate and Mediterranean Spain to characterize species' trophic roles and investigate whether these roles are linked to specific functional traits. We characterized a species' role by measuring four species‐level network metrics (i.e., normalized degree, specialization, closeness, and betweenness centrality; Dormann et al. 2009). We combined these metrics into a single centrality metric to describe the general species' trophic role. We then related species' centrality with a compiled dataset of functional traits hypothesized to influence a scavenger function. We hypothesized that scavengers with functional traits that facilitate the detection and access to carcasses, such as large home ranges, keen eyesight, or sense of smell, are likely to have more of a central role in the networks since they will be able to rapidly locate carcasses of different species irrespectively of their body size or habitat. In contrast, we expected species with traits that limit the detection and access to carrion of different species, such as small home ranges, to be peripheral in the networks. This study provides information about the diverse roles of scavengers by identifying species that contribute more to network cohesion, species that may be more vulnerable to changes in the community, and the functional traits that characterize these species. This information is relevant for defining target species for conservation efforts to maintain scavenger communities and ecosystem functions and services.

## 2 Methods

### 2.1 Study Area

**FIGURE 1:**
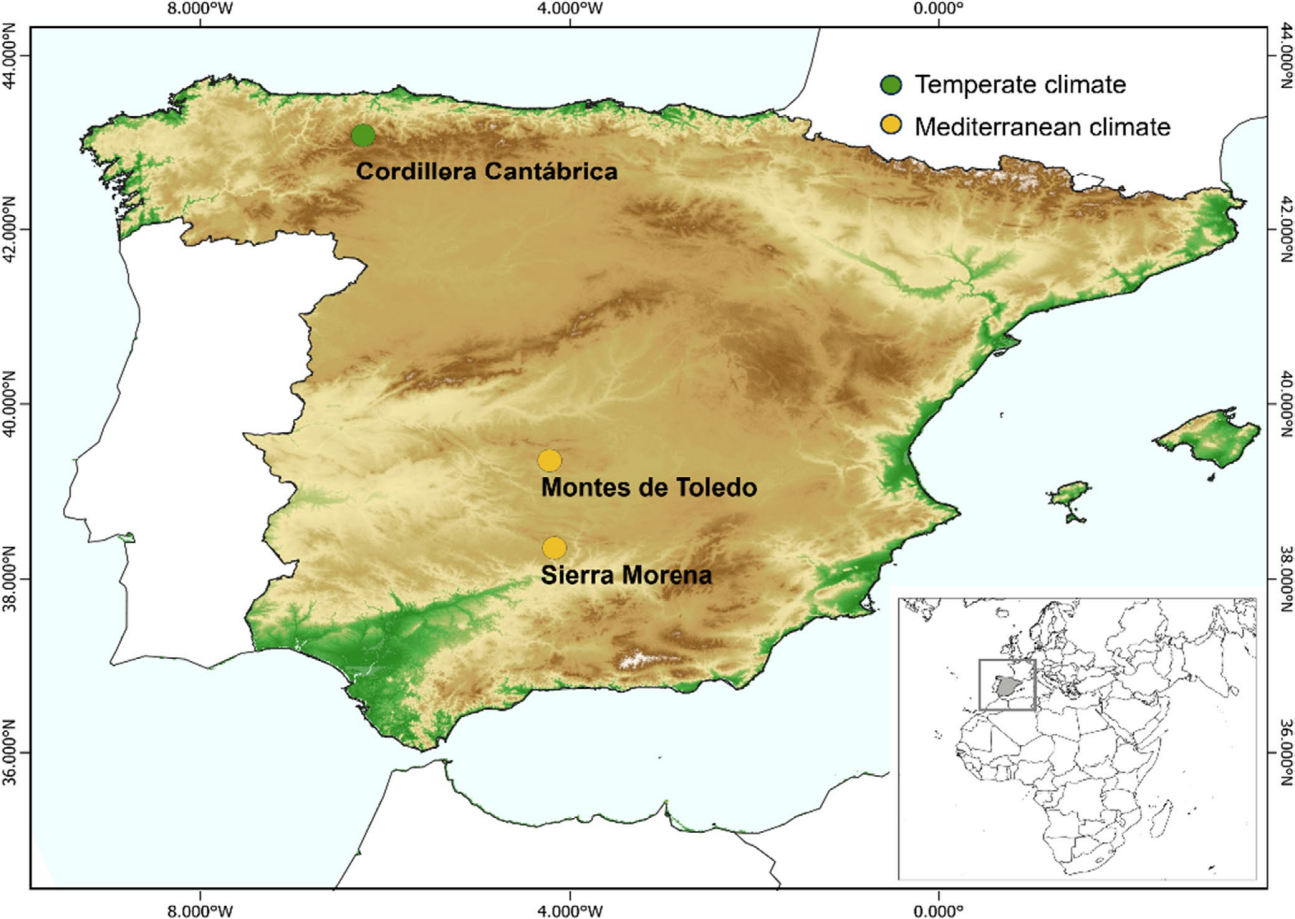
Vertebrate scavenger networks were monitored in temperate (Cordillera Cantábrica; green dot) and Mediterranean (Montes de Toledo, Sierra Morena; yellow dots) Spain. The map colors indicate the different elevations throughout the countries, ranging from 0 (green) to > 3000 m (dark brown).

We analyzed vertebrate scavenger networks in three study areas in north temperate and central and south Mediterranean Spain (Figure 1). Cordillera Cantábrica, in the north of Spain, belongs to the Atlantic biogeographical region, which covers ~11% of the country, and it is characterized by a temperate climate. Sierra Morena and Montes de Toledo, in central and southern Spain, are part of the Mediterranean region, which is present in ~87% of the country and has a dry season during summer (AEMET 2011). Using these three study sites enabled us to capture the diversity among vertebrate scavenger communities and to discern the varying roles of the same species across different locations.

The temperate ecosystem covers a wide altitudinal range (i.e., from ∼400 to > 1200 m.a.s.l.) and encompasses diverse habitats, including oak and beech woodlands, pastures used for extensive livestock breeding, and rocky outcrops. The Mediterranean ecosystems consist mainly of crops and *dehesas* (i.e., traditional agroecosystems characterized primarily by open woodland with extensive livestock grazing), Mediterranean forests, and pine plantations, with altitudes ranging between ∼500 and ∼1000 m.a.s.l. (Mateo‐Tomás et al. 2017; Pardo‐Barquín, Mateo‐Tomás, and Olea 2019). These study areas are home to most vertebrate scavengers in the Iberian Peninsula, including apex predators, obligate scavengers, and generalist species. The Cordillera Cantábrica is the only area containing the mammalian apex predators brown bear (*Ursus arctos*) and Iberian wolf (*Canis lupus*), thus displaying a more complete trophic community than the Mediterranean sites, which instead have a wider variety of avian species. Griffon (*Gyps fulvus*) and cinereous vultures (*Aegypius monachus*) were more abundant in Mediterranean ecosystems, whereas the Egyptian vulture (*Neophron percnopterus*) was more abundant in the temperate study area (De la Puente, Moreno‐Opo, and González 2007; Del Moral 2009; Del Moral and Molina 2009). Red foxes (*Vulpes vulpes*) are common in all three study areas, wild boars (*Sus scrofa*) are more abundant in the Mediterranean than in the temperate ecosystem and the opposite occurs with corvids (Table S1).

### 2.2 Scavenger Interaction Network

Scavenger interactions were sampled in the three study areas for three years (2011–2013). We used camera traps (one battery powered, IR and motion‐triggered camera of brand Ltl Acorn or ScoutGuard per carcass) to monitor the consumption of 180 carcasses consisting of remains of four free‐living, wild ungulate species obtained from sport hunting or culling. Cameras were placed near the carrion (4–8 m) just after its disposal and took three pictures every minute if movement was detected both at day and night, using no glow infrared sensors to minimize disturbance. The study included 68 wild boar carcasses, 89 red deer (*Cervus elaphus*), 22 chamois (*Rupicapra rupicapra*), and one roe deer (*Capreolus capreolus*) carcass. These figures reflect the abundance and distribution of these wild ungulates, and the availability of their carcasses mediated by hunting across the three study areas. The inclusion of different carcass species allowed us to analyze a network based on species‐to‐species interactions, providing a new perspective compared to previous studies that focused on species‐to‐carcass interactions (e.g., Sebastián‐González et al. 2020).

The carrion was obtained from hunters or rangers and consisted of entire corpses, body remains, and guts. All carcasses were monitored either immediately after hunting or following their relocation, with collaboration from hunters and rangers in the three study areas. In Cordillera Cantábrica, we monitored remains directly after they were generated, as we accompanied rangers and hunters during their hunting activity. In Montes de Toledo and Sierra Morena, we collected remains at processing sites right after hunting and then relocated them within the same hunting areas to mimic the spatial segregation from hunting activities. The number of monitored carcasses and remains was 72 in Cordillera Cantábrica, 61 in Montes de Toledo, and 47 in Sierra Morena. In Cordillera Cantábrica, carcasses of all four ungulate species were monitored, but in Montes de Toledo and Sierra Morena only carcasses of red deer and wild boar were used, since chamois is absent and roe deer is scarce. Due to the natural origin of the carcasses, we couldn't place the same amount of each carcass species in each study site. However, the network metrics we used in the analysis were standardized to allow comparison across networks (see Appendix S1 for details).

Habitat characteristics play a crucial role in shaping the scavenger community at a carcass by influencing how easily it is discovered by each species (Pardo‐Barquín, Mateo‐Tomás, and Olea 2019). To account for different habitat types and better reflect natural carcass distribution, we monitored about half of the carcasses in open land (53% in Cordillera Cantábrica, 55% in Sierra Morena, 47% in Montes de Toledo), and the remaining half in shrubland or forest sites. Carrion was weighted in the field after their generation and/or deposit and just before the placement of the camera. Carcass mass was obtained using one portable gauge, while entire corpses' weight was estimated using corporal measures and available references on the species' sizes. The carcasses' initial weight ranged from 2 to 104 kg and were monitored during all seasons of the year until complete consumption or until only bones and skin remained. Although we did not include carcass size in our analysis to simplify the study and increase sample size, the broad range of sizes should help randomize this variable and reduce potential bias.

A species was considered to have scavenged a carcass if the pictures clearly showed consumption. If consumption was suspected but not directly observed (e.g., an individual was recorded closely inspecting a carcass), we inferred the consumption when the species was confirmed to be feeding on other carcasses in our study. We recorded the minimum abundance of species per carcass, defined as the maximum number of individuals of the species simultaneously appearing on the same picture (see Mateo‐Tomás et al. 2017 for additional details). Scavenging individuals were identified manually in all pictures by at least three people to confirm the species. In the Cordillera Cantábrica, where two *Martes* species overlap, we assigned only the genus due to challenges distinguishing species by fur color in black‐and‐white night photos.

Based on these camera records, we constructed quantitative scavenger–species interaction networks at each location, represented by matrices where rows denote carcass species and columns indicate scavenger species. The matrix entries quantify the frequency of interactions between scavenger and carcass species (i.e., the proportion of carcasses of a given species visited by each scavenging species), forming bipartite networks where two distinct groups—scavengers and carcasses—interact exclusively with each other without intragroup interactions (Costa et al. 2007).

### 2.3 Scavenger species' Life‐History Traits

For the analysis, we selected a set of 13 morphological, life‐history, and functional traits that had information for at least 70% of the species, did not correlate with each other and are hypothesized to affect scavenger species' trophic roles (Table 1). Scavenger functional traits were obtained from the databases AVONET (Tobias et al. 2022), EltonTraits (Wilman et al. 2014), Amniota (Myhrvold et al. 2015), and PanTHERIA (Jones et al. 2009), as well as the online sources Animal Diversity Web (Myers et al. 2006) and Birds of the World (Billerman et al. 2022). We also considered the scavenger species conservation status at national level (RD 139/2011; Boletín Oficial Del Estado 2011), which is related to species' abundance and distribution (IUCN Standards and Petitions Committee 2022) and can therefore explain their role in the networks.

**TABLE 1:** Description of the functional traits used in this study and hypotheses describing the expected relationship between species' traits and species trophic role in the networks. The conservation status of each species as stated by the Spanish law (BOE 2011) was also considered.

Trait	Description	Hypothesis	Source
Omnivory level	Variety of food resources exploited by species (e.g., fruit, seed, carcass). Ranges from 0.1 (specialists, i.e. species feeding on only one type of resource) to 1 (generalist, i.e. species feeding on all types of resources assessed)	We expect species with a high omnivory level to be more central because they are more likely to opportunistically consume carcasses from different species than species more specialized in a food resources other than carrion (e.g., some predators specialized in hunting concrete prey species like birds, rabbits or rodents)	Selva et al. (2019 **)**
Body mass	Natural logarithm of the mean adult weight (in grams)	Large species can eat more quantity, and thus could interact with more carcasses. Through exploitation competition, they can prevent other species from feeding on some of them. These factors would make large species more central, given that they would have a larger normalized degree and would displace other species to peripheral roles. Additionally, large‐bodied species can be more efficient scavengers able to survive on body reserves in the periods between discovering carrion	Ruxton and Houston (2004 **)**, Mateo‐Tomás et al. (2017 **)**
Extremities–body length ratio	Ratio of extremities length (mm) over body length (mm) The extremities of birds are measured as the length from the carpal joint (bend of the wing) to the tip of the longest primary on the unflatten wing. For the extremities of mammals, we used the shoulder height. Body length was the distance from the tip of the beak or the snout to either the opening of the cloaca or to the anus or tail base for birds and mammals respectively	Extremities length is used as a proxy for movement efficiency, which is necessary for scavengers to cover large areas and to increase their encounter rate with an a priori unpredictable resource like carcasses. For birds, hand‐wing index (i.e., wing length divided by Kipps distance, a measure of wing elongation) is a measure of flight efficiency, and it is positively correlated with wing length (*r*_P_ = 0.67). For terrestrial animals, longer limbs improve walking and running economy. To allow comparisons across scavengers of different sizes, we normalized extremities length by dividing it by the body length We expect a species with larger extremities‐body length ratios to be more central due to their increased carcass encounter rate, promoted by their increased movement efficiency sustained by their longer limbs	Ruxton and Houston (2004), Pontzer (2007), Sheard et al. (2020)
Home range	The natural logarithm of the individual home range (in km^2^)	Large home ranges can promote the discovery of carcasses by a species and may lead to an increased number of interactions with carcasses due to shorter detection times, and thus, to a larger centrality	Ruxton and Houston (2004 **)**, Gutiérrez‐Cánovas et al. (2020 **)**
Habitat breadth	Index of habitat specialization based on patterns of species co‐occurrence calculated by Ducatez	Species with a larger habitat breadth occur in diverse sites with more species that can scavenge on carrion, thus can visit more carcasses. Then, we expect to make them more central to the network	Ducatez, Tingley, and Shine (2014)
Activity	Species' daily activity as: diurnal (active during daytime), nocturnal (active during the night), cathemeral (irregular activity at any time during night or day), diurnal and crepuscular (active at dawn and dusk and during the day), and nocturnal and crepuscular (active at dawn and dusk and during the night)	Scavenger species active during both day and night could have more opportunities to locate and consume carrion, thereby increasing their centrality	Butler and du Toit (2002), Gutiérrez‐Cánovas et al. (2020 **)**
Foraging behavior	The way species forage. Social: in groups, species acquire information from conspecifics. Solitary: species don't profit from the acquisition of information from other individuals of its species	Social species can profit from the transfer of information between conspecifics to locate carcasses, which we expect to increase their centrality to the network compared to solitary species	Kane et al. (2017 **)**
Class	The categorical variables' olfaction' (high/low) and ‘mobility’ (flying/non‐flying) are represented by the variable' class' (i.e., bird/mammal), which expresses the same information as these two traits. All mammals were terrestrial and had good olfaction, whereas all birds could fly and had less acute olfaction	High olfactory acuity increases the detection rate of scavenger species. We thus expect species with acute olfactory abilities to be central to the network	Ruxton and Houston (2004 **)**, Kane et al. (2017 **)**
		Aerial flight allows species to move over larger areas efficiently, thus increasing their capacity to find carrion, which we expect will increase their centrality. On the other hand, flying species may struggle to access carcasses of species inhabiting areas of dense vegetation	
Predation	Predatory behavior: top‐predator, meso‐predator or non‐ predator	Top‐predators show high specialization in carrion consumption, which allows them to access all types of carcasses and thus increase their centrality; at the same time, they can also exclude other species from carcasses through exploitation competition, thus displacing them to peripheral roles and increasing their own centrality	Moleón et al. (2015), Gutiérrez‐Cánovas et al. (2020)
Sight	High or low visual acuity	Species with good visual abilities, such as vultures, can locate carrion efficiently from above while flying at heigh altitudes, so they can rapidly detect carcasses over large areas, becoming thus more central to the network	Houston (2001 **)**
Noisiness	Use of vocalizations while foraging	Species that use vocalization while foraging act as indicators of carrion. By giving cues to their conspecifics and other species about the location of carcasses, they interact with carcasses that are visited by more scavengers, making them more central	Selva et al. (2019 **)**
	Noisy species vocalize loudly while foraging. Quiet species forage silently		
Species conservation characteristics			
Conservation status	The conservation status of species as defined by the Spanish law: ‘3’: Endangered; ‘2’: Vulnerable; ‘1’: Special protection (LESPRE); ‘0’: No particular protection	Here, the conservation status is used as a proxy for species abundance and/or distribution. We expect abundant species to be more central to the networks, since it is more likely they interact in larger numbers or more often with carrion	Laurindo et al. (2020 **)**

### 2.4 The Trophic Role of Scavengers

To measure species' trophic role in the network, we used species‐level centrality metrics that characterize the species' position in the network (Cirtwill et al. 2018). We calculated four metrics using the ‘bipartite package’ (version 2.18) in R (Dormann et al. 2009): (i) normalized degree (ND), (ii) specialization (d′), (iii) closeness centrality (C_c_), and (iv) betweenness centrality (C_b_). These metrics describe whether a species is central or peripheral in the network. Species with a large normalized degree could be important to maintain ecosystem functions such as nutrient recycling because they find and scavenge on most carcasses. The specialization d′ metric describes the level of specialization of a scavenger species using carrion, where being a generalist means interacting with carrion species that are abundant, while being a specialist means using few and rare resources (Dormann et al. 2009). Closeness centrality describes how distant a scavenger is to all other species in the community and indicates how rapidly a species is likely to influence the overall community if, for example, there is a perturbation, such as the loss of that species (Estrada and Bodin 2008). Betweenness centrality characterizes scavenger species that act as a ‘bridge’ for energy transfer within the network (Cirtwill et al. 2018), which, if removed, would disconnect the network into multiple components. Further details for the metrics' interpretation and calculation can be found in Appendix S1.

The four metrics described before were combined using a principal component analysis (PCA) to obtain a single centrality metric summarizing the information on the trophic role of scavenger species at each site (Medeiros et al. 2018; Moulatlet, Dáttilo, and Villalobos 2023). The first principal component (PC1) negatively correlated with the metrics that indicate higher centrality (i.e., normalized degree, closeness centrality, and betweenness centrality) and positively correlated with specialization, which indicates lower centrality. To facilitate the interpretation and ranking of species based on their centrality, species' variable loadings and PC scores were transformed by multiplying them by −1 (i.e., ‘centrality score’ hereafter). Since metric values were computed separately for each species in each network, those species present in multiple networks had more than one centrality score.

### 2.5 Data Analysis

To explore the relationship between functional traits and the trophic role of vertebrate scavengers in the networks, we fitted generalized linear mixed models (GLMMs) and performed a model selection analysis by exhaustive screening to evaluate all possible combinations of predictor variables using the R package ‘glmulti’ (version 1.0.8; Calcagno and de Mazancourt 2010). Species centrality scores for each network were modeled as the response variable using a Gaussian distribution. In the first step, we selected the optimal structure of the random component using REML estimators to compare models with different random structures. Both ‘network ID’ and ‘class’ were used for the analysis as random components (see Appendix S1 for information on the variable selection process).

Next, we searched for the optimal fixed structure of the model by implementing a multi‐model inference approach with the R package ‘glmulti’ (Calcagno and de Mazancourt 2010). We evaluated all possible combinations of scavengers' functional traits, incorporating as random effects ‘network ID’ and ‘class’, because they showed the best fit in the previous step. Models were ranked by Akaike's information criterion corrected for small sample sizes (AICc), with the best models selected for model‐averaging to generate averaged parameter estimates, identifying key variables by their summed evidence weights across models (Calcagno and de Mazancourt 2010).

A two‐sample Wilcoxon rank sum test was also performed to evaluate whether the difference in centrality scores between obligate and facultative scavengers was statistically significant, using a significance threshold of *p*‐value < 0.05. This conventional standard was chosen to provide balance between the risk of Type I and Type II errors. Additionally, we used a Kruskal–Wallis test to explore the differences in centrality among species with different conservation statuses. All data analyses were performed in R version 4.3.1 (RStudio Team 2022).

## 3 Results

### 3.1 Scavenger Characteristics

**FIGURE 2:**
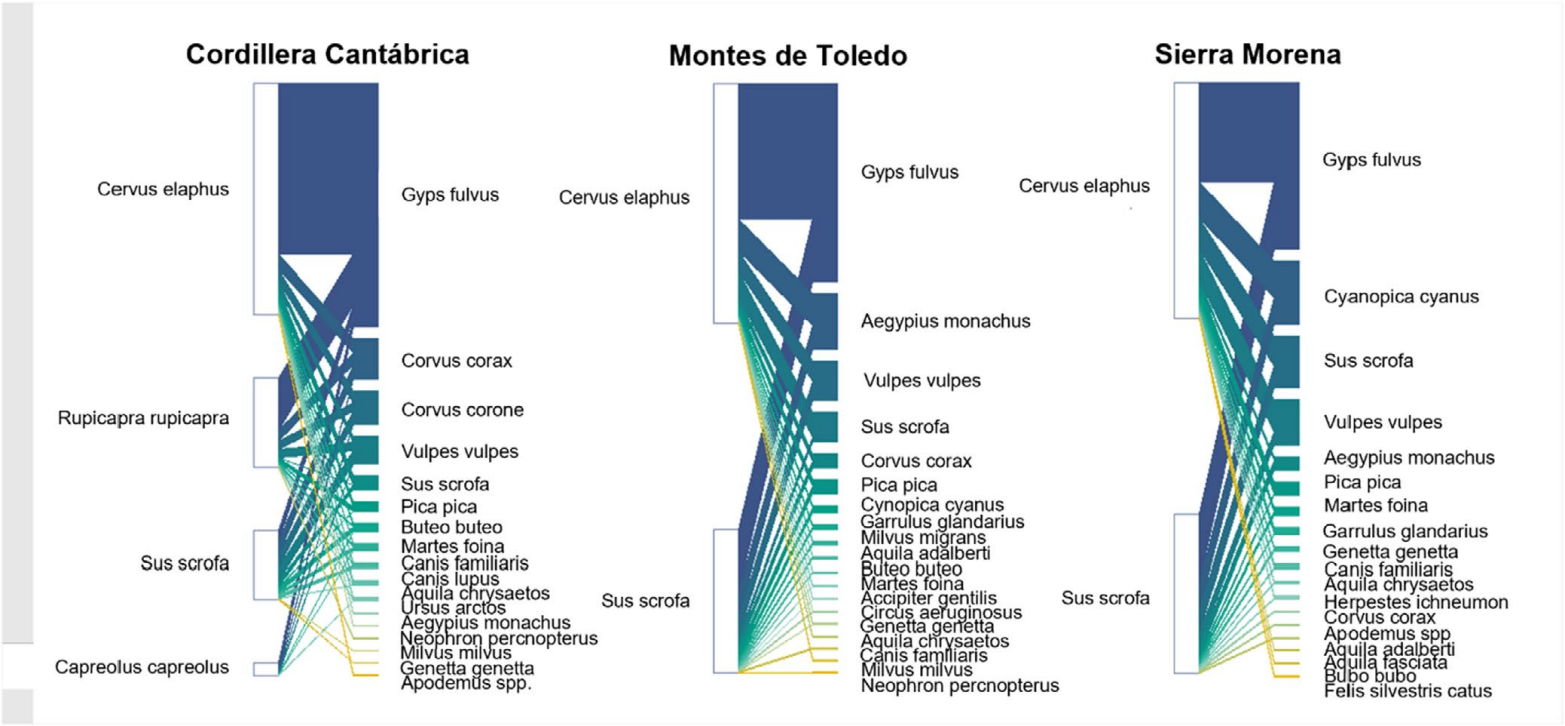
Scavenger interaction networks in each study area: Cordillera Cantábrica, Montes de Toledo, and Sierra Morena. The studied scavenger networks are visualized as bipartite networks, with scavenger species on the right and carrion species on the left of each plot. The lines between the two groups represent trophic interactions (a scavenger species feeding on a carcass species). The width of the trophic interaction represents the frequency with which the scavenger species fed on a carcass species.

In the three scavenger networks—Cordillera Cantábrica, in temperate Spain, and Montes de Toledo and Sierra Morena in Mediterranean Spain—17, 21, and 18 vertebrate scavenger species were recorded feeding on wild ungulate carcasses, respectively (Figure 2; Table S1 in Appendix S1). Birds accounted for 65% of the scavenging species recorded (*n* = 34). Three obligate scavengers were recorded in all the study areas (cinereous, griffon and Egyptian vultures), although their abundances differed among sites (Del Moral 2003, 2009; Del Moral and Molina 2009; De la Puente, Moreno‐Opo, and González 2007). The two mammalian apex predators present in the Cordillera Cantábrica, namely, gray wolf and brown bear, were recorded at the monitored carcasses.

The home range of scavenger species ranged between 0.01 and 46,735.76 km^2^ (mean: 4501.02, median: 9.32, SD: 12,192.32), the mean body mass between 0.02 and 205.80 kg (mean: 13.58, median: 2.01, SD: 35.92), and the extremities and body length ratio ranged between 0.25 and 0.84 m (mean: 0.63, median: 0.66, SD: 0.16; Table S11). Although these values are not specific to the study sites, they provide a general framework for understanding typical species characteristics. Scavengers had an average omnivory level of 35%, with cinereous and griffon vultures displaying the lowest levels at 10%, indicating that, although carcasses comprise their main food source, they still occasionally consume other resources (e.g., plant material; Fernández 1998, Yamaç and Günyel 2010). Most scavengers exhibited low olfactory capabilities (61%). Scavengers typically engaged in solitary foraging (57%) and functioned as meso‐predators (midranking predators in food webs, 65%; Prugh et al. 2009). Their foraging activities predominantly occurred during daylight hours (61%), taking advantage of their keen eyesight (83%). A considerable proportion of species (65%) were in silence while foraging, a trait observed across all mammalian species, according to the compiled database.

In total, 13 species were under special protection by Spanish law. The endangered species recorded were the Spanish imperial eagle (*Aquila adalberti*), red kite (*Milvus milvus*), and brown bear. Additionally, the networks encompassed two species legally classified as vulnerable: the Egyptian and cinereous vultures. The remaining eight protected species are included in the Spanish list of species under special protection while being classified as Least Concern by the Red List assessment of the International Union for Conservation of Nature (IUCN 2021).

### 3.2 Scavenger Trophic Roles

**FIGURE 3:**
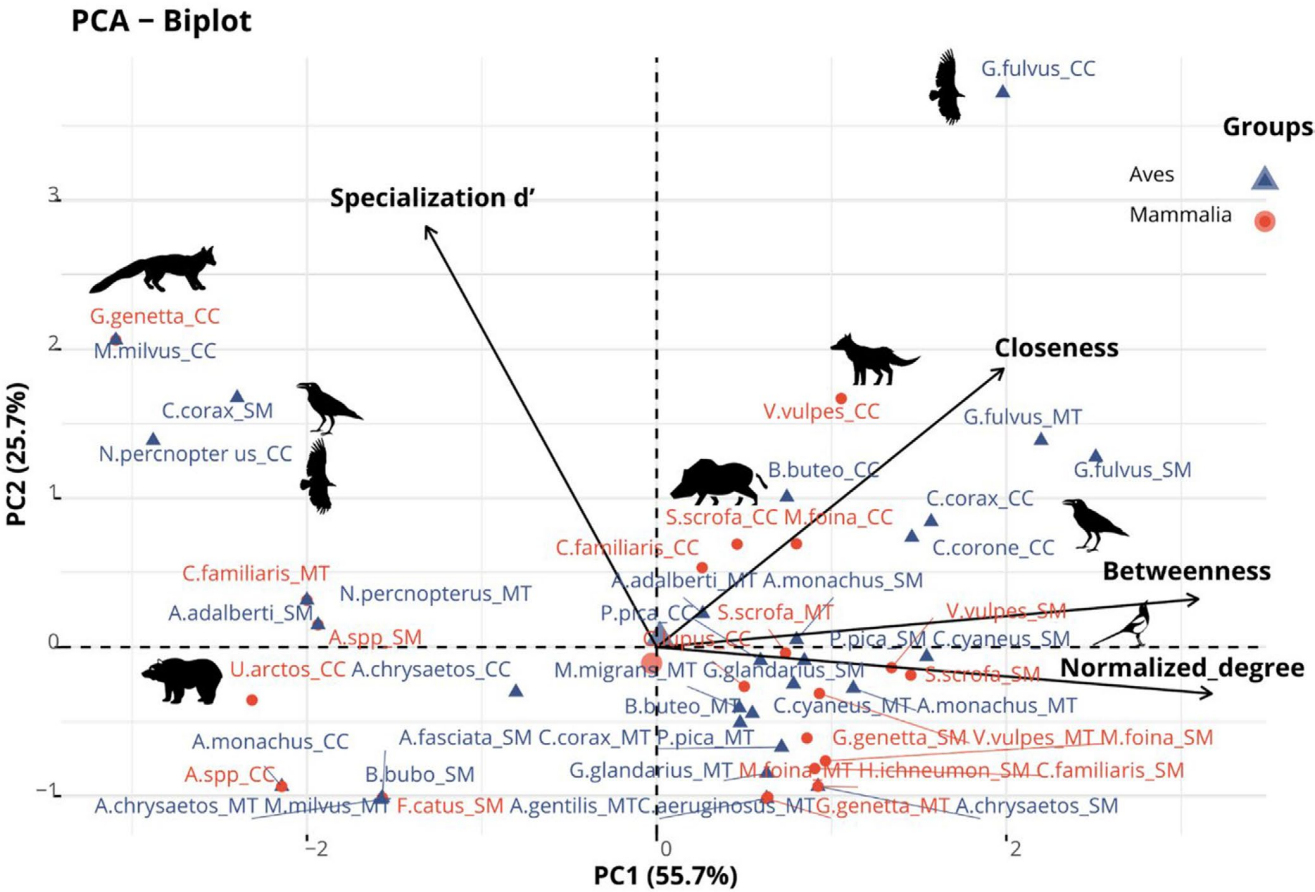
Principal component analysis biplot showing the loadings of four centrality metrics normalized degree, betweenness centrality, closeness centrality, and specialization d′ (arrows). The position of the individual observations for each species in each network is shown (see Table S15 for the legend of the abbreviations of the species' names). Red points are mammals and blue triangles are birds. CC, Cordillera Cantábrica; MT, Montes de Toledo; SM, Sierra Morena. Icons taken from The Noun Project https://thenounproject.com/. Created by Ricardo Moreira, Philipp Lehmann, Evgeni Moryakov, Aleks, Tom Fricker, Travis us, Andreas Reich, and Abdul Basiith.

The four metrics revealed a high diversity in the species' position in the network (Table S13) and different contributions to the centrality score. After running the PCA, the first PCA axis (PC1) explained 55.68% of the total variance, while the second one (PC2) explained 25.67% (Figure 3). PC1 scores ranged between −3.1 and 2.5 (mean: 0.0, median: 0.6). Normalized degree exhibited the highest positive loading value at 0.63, indicating a strong positive association with the first principal component (PC1). This suggests that variations in this metric play a significant role in explaining the underlying centrality patterns. Betweenness centrality also displayed a substantial positive loading of 0.61, indicating its relevance in capturing an individual's centrality. Closeness centrality had a positive loading of 0.39, indicating its moderate contribution to the first principal component. On the other hand, specialization d′ showed a negative loading of −0.26, reflecting its negative contribution to centrality. We used PC1 scores (i.e., centrality scores) to represent the centrality of the 27 scavenger species, where the highest values represent more central species in a network and the lowest values characterize peripheral species. Specialization d’ was better represented by PC2, with a contribution of 0.83 (Figure 3, Table S14).

The species with the highest overall network centrality across all sites was the griffon vulture, followed by the raven in Cordillera Cantábrica and the azure‐winged magpie in Montes de Toledo, indicating a potential key role in maintaining the scavenger network structure and function, by feeding on more carcasses irrespective of their species. Based on their low centrality scores, the red kite, common genet, brown bear, and Egyptian vulture in Cordillera Cantábrica displayed low centrality (Table S16). Furthermore, the Egyptian vulture was among the most peripheral species for all three networks. Contrastingly, species like the common raven exhibited high and low centrality across ecosystems. Cordillera Cantábrica had the species with the highest centrality (centrality score = 1.57), while Sierra Morena exhibited the species with lowest centrality (centrality score = −2.40) (Table S16).

Some species had very different roles depending on the network to which they belonged. The cinereous vulture had a peripheral role in Cordillera Cantábrica (centrality score: −2.14), where the species is not resident but vagrant (De la Puente, Moreno‐Opo, and González 2007). Contrastingly, this vulture exhibited a rather central role in Montes de Toledo and Sierra Morena (1.12 and 0.80, respectively), where the species has some of its breeding strongholds. Similarly, in agreement with its uneven distribution (Del Moral 2003), the raven had very contrasting roles in different networks, with the highest score of 1.57 in Cordillera Cantábrica, and lower values in Montes the Toledo (0.71), and Sierra Morena (−2.40). The common genet was more central in the southern networks (0.63 in Montes de Toledo and 0.86 in Sierra Morena) and peripheral in Cordillera Cantábrica (−3.09). The golden eagle was peripheral in Cordillera Cantábrica and Montes de Toledo (−0.80 and −1.58, respectively), while in Sierra Morena, it was more central (0.92), as did the dog, with a positive score in Sierra Morena and negative scores in Cordillera Cantábrica and Montes de Toledo.

**FIGURE 4:**
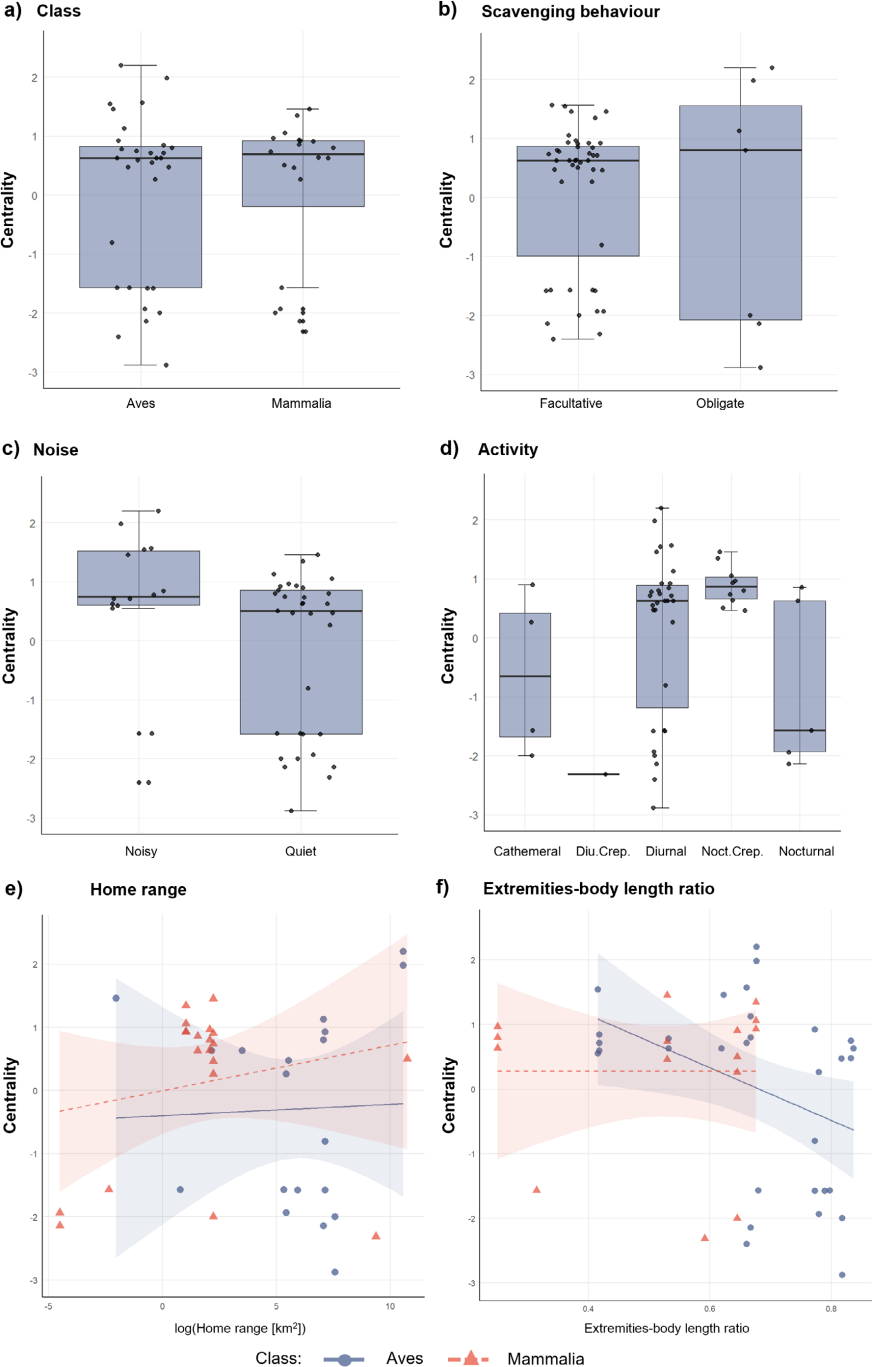
Variation in scavenger centrality (PC1 score). (a and b) explore PC1 centrality of birds and mammals (a), and that of obligate and facultative scavengers (b). c–f represent the four important variables selected by the best models: (c) noise, (d) activity, where ‘d_c’ stands for diurnal and crepuscular, ‘d’ for diurnal, ‘n_c’ for nocturnal and crepuscular, ‘n’ nocturnal’ and ‘c’ for cathemeral; (e) shows extremities‐body length ratio and (f) the home range. In plots (e) and (f), red shows the relationship between mammals' centrality and functional traits, while blue shows the same information but for birds.

Birds and mammals did not show a significant difference in centrality (Wilcoxon's test *W* = 342.5, *p*‐value = 0.95, Figure 4a), although different trends were observed between the two classes for certain traits.

### 3.3 Functional Traits Explaining Scavenger Trophic Roles

According to the best models, species with larger home ranges, larger extremities−body length ratios, and that are active both at night and crepuscule were central to the analyzed networks (Table 2; Figure 4). In contrast, scavengers that are quiet while foraging, as well as species that are active both during the day and the crepuscule, or only at night, assumed a peripheral role in the networks (Figure 4).

**TABLE 2:** Averaged parameter estimates of the best models explaining the scavenger species centrality using network and class as random intercepts. The most important variables were extremities–body ratio and home range, which positively influenced species' centrality, and the categorical variables noise and activity.

Functional Trait	Estimate	Importance
Extremities–body ratio	0.591	1.0
Home range	0.013	1.0
Noise–quiet	−3.047	1.0
Activity–diurnal	−0.403	1.0
Activity–diurnal and crepuscular	−1.456	1.0
Activity–nocturnal	−4.293	1.0
Activity–nocturnal and crepuscular	1.607	1.0

Apex predators and obligate scavengers were among the species displaying a central role and had the largest home ranges, i.e., the griffon vulture in all three networks, the wolf in Cordillera Cantábrica, and the golden eagle in Sierra Morena. Three species with the largest extremities−body length ratios but that were also peripheral were the Egyptian vulture and red kite in Cordillera Cantábrica and Montes de Toledo and Bonelli's eagle in Sierra Morena. Regarding their omnivory level, generalist species like the wild boar, the red fox, the beech marten, and the wolf were characterized by their nocturnal and crepuscular activity pattern. All four species displayed centrality to some degree (centrality score > 0).

The size of the home range showed a stronger positive relationship with centrality in mammals than in birds (Figure 4e), while the species extremities−body length ratio explained centrality in birds but not in mammals (Figure 4f). Obligate scavengers showed a slightly higher centrality than facultative scavengers, but this difference was not significant (Wilcoxon's test: *W* = 144, *p*‐value = 0.336, Figure 4b; see Table S17).

**FIGURE 5:**
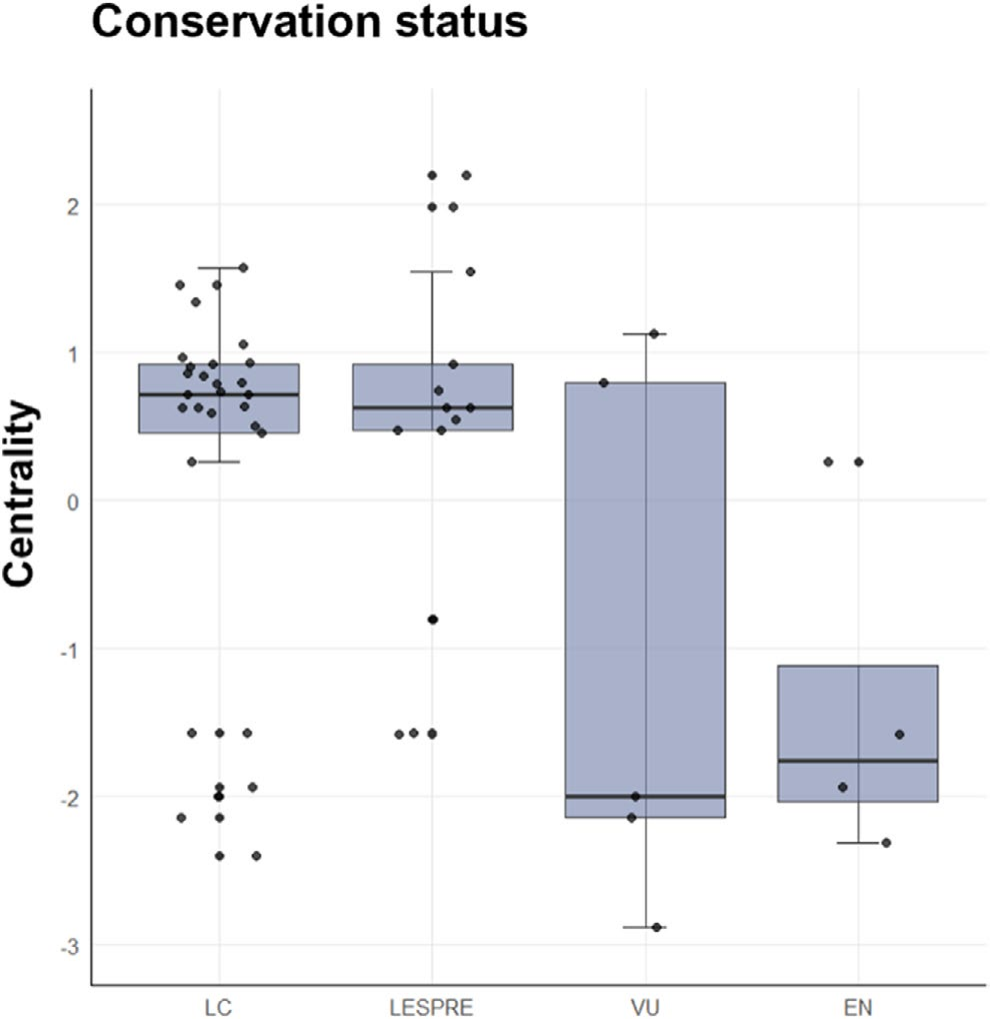
The scavenger species conservation status. Vulnerable (VU) and endangered (EN) species by the Spanish law (BOE 2011) had lower centrality than the species listed at the lowest categories of protection (i.e., LESPRE) and least concern (LC).

The Kruskal–Wallis test revealed significant variation in the centrality of species with different conservation status (*χ* ^2^ = 9.19, *p*‐value = 0.03, df = 3). Species of least concern (LC) or those listed in the lowest protection level in Spain (i.e., the Spanish list of wild species under special protection regime, LESPRE) tended to have high centrality. Contrastingly, the most threatened species tended to be peripheral to their networks (Figure 5). Among the species categorized as vulnerable with low centrality were the Egyptian vulture in Cordillera Cantábrica and Montes de Toledo and the cinereous vulture in Cordillera Cantábrica. In contrast, cinereous vultures in Montes de Toledo and Sierra Morena were more central. Endangered peripheral species were the Spanish imperial eagle, the brown bear, and the red kite.

## 4 Discussion

Our findings suggest that the trophic role of scavenger species is intricately tied to their functional traits, which can give predictive insights into a species' functioning within a community. Although these trends depend on species abundance and dominance, scavengers with large home ranges, nocturnal, crepuscular activity patterns, or vocalizations during foraging tend to emerge as central figures within scavenger networks. These functional traits serve as indicators of key species that warrant protection to sustain or enhance the ecological function of scavenging, given that they play a role in maintaining network cohesion by feeding on most carcasses. Central species increase the number of feeding links, acting as cohesive bridges that bind the various components of the network together (Bascompte 2009; Moleón et al. 2015). The significance of these central species becomes evident when considering the potential consequences of their disappearance—where the entire network could rapidly collapse (Bascompte 2009). Besides, changes in the abundance of central species can disrupt scavenging activities of other species due to the fallout of facilitative processes, such as large species opening carcasses for smaller scavengers or smaller birds facilitating the location of carcasses to other scavengers (Selva et al. 2003; Kane et al. 2014; Moreno‐Opo and Margalida 2019; Olea, Iglesias, and Mateo‐Tomás 2022). Therefore, the protection of these key species may be paramount for the preservation of scavenging networks and the ecological functions they support.

Home range size was one of the most useful traits for determining central roles. Species with large home ranges, such as obligate scavengers (e.g., > 46,000 km^2^ for griffon and cinereous vultures; Morales‐Reyes et al. 2016) or apex predators, are indeed related to higher carrion consumption rates through an increased capacity to detect carcasses over vast territories as opposed to the smaller foraging areas of facultative scavengers (Ruxton and Houston 2004; Kane et al. 2017; Gutiérrez‐Cánovas et al. 2020). In fact, long‐distance foragers like griffon vultures (a central species in the networks here analyzed; Figure 3; Table S16) can connect local assemblages within the metacommunity, supporting scavenger diversity and functions across space (Mateo‐Tomás, Olea, López‐Bao, et al. 2019; Mateo‐Tomás, Olea, Selva, et al. 2019). Additionally, species with large home ranges can contribute to the transport of nutrients over large areas, distributing nutrients within and across ecosystems (Schlichting et al. 2019).

In contrast to previous expectations, obligate scavengers were not significantly more central than facultative scavengers in our scavenger networks. Vultures are the only obligate scavengers among extant vertebrates (Ruxton and Houston 2004). Functional traits like soaring flight, keen eyesight, and large body mass allow vultures to efficiently search for unpredictable carrion food resources over vast areas, thereby overcoming the constraints imposed by an obligate scavenging lifestyle. These characteristics increase the ability of vultures to find and access carrion and would explain their central roles in the networks as well as their scavenging efficiency (Mateo‐Tomás et al. 2017). However, vultures are often not the functionally dominant scavengers; instead, facultative scavengers, like apex predators and wild boars, dominate carrion consumption where vultures are absent or scarce (Mateo‐Tomás et al. 2017). This can explain why not all vulture species acted as central species in the study areas, emphasizing the importance of both obligate and facultative scavengers in ecosystem functioning. For example, the peripherical roles of the Egyptian vulture in Montes de Toledo and the cinereous vulture in Cordillera Cantábrica could be explained by their low density in these sites (De la Puente, Moreno‐Opo, and González 2007; Del Moral and Molina 2009). Contrastingly, the griffon vulture, which was abundant and widespread across the three study areas, showed a major central role across the scavenger networks analyzed.

Drastic declines in several vulture populations across the world (Prakash et al. 2003; Oaks et al. 2004; Ogada et al. 2016) have drawn attention to the many threats that vultures face and the implications of their loss (Ives et al. 2022). Vulture declines may have major implications for ecosystem functioning. On one hand, the rate at which energy and nutrients flow through food webs might decrease (DeVault, Rhodes Olin, and Shivik 2003; DeVault et al. 2016; Mateo‐Tomás et al. 2017). On the other hand, the loss of vultures (and other facultative apex scavengers like apex predators, e.g., lions; Mateo‐Tomás et al. 2017) can result in a mesoscavenger release, where less efficient scavengers increase in abundance in the absence of competition from more efficient obligate scavengers (O'Bryan, Holden, and Watson 2019). For instance, in the Indian subcontinent, dogs (*C. l. familiari*s) increased significantly following major vulture declines (Prakash et al. 2003; Markandya et al. 2008), resulting in an increase in rabies transmission due to dog bites (Markandya et al. 2008). Indirect effects of this mesoscavenger release can therefore have a negative impact on ecological functions and services, such as disease control, pest prevalence, or invasion potential (O'Bryan, Holden, and Watson 2019), emphasizing the importance of vultures and apex predators in ecosystems.

The ratio between the extremities and body size, a proxy for movement efficiency, negatively influenced species' centrality, especially for birds. The effect of large extremities on the centrality of mammals was unclear, with the centrality of species characterized by large ratios ranging from peripheral (e.g., brown bear) to rather central (wolf). This weak relationship would suggest that the general inefficiency of terrestrial mammals as scavengers (Ruxton and Houston 2004), even among larger species with larger home ranges, could not be attributed to their terrestrial mobility. However, this weak relationship observed in mammals in our study areas may be affected by the relatively low abundance of the brown bear, a species officially listed as endangered (Boletín Oficial Del Estado 2011). That birds with large extremities tended to be more peripheral can be explained by species abundance and distribution, where peripheral birds with large extremities included species listed as threatened species by the Spanish law (i.e., vulnerable and endangered; Boletín Oficial Del Estado 2011) including the Egyptian vulture, the red kite, and the Spanish imperial eagle. The peripheral role of the eagle owl (*Bubo bubo*), an abundant bird of prey in the south of Spain, may be related to the species being a rare scavenger (Mateo‐Tomás et al. 2015). In addition, hierarchy/subordinate behavior could further explain peripheral positions of species with long extremities (Moreno‐Opo, Trujillano, and Margalida 2020). For example, Egyptian vultures are dominated by other vultures, e.g., the griffon vulture, relegating it to be a secondary consumer that can only access carcasses after dominant vultures have left (Meretsky and Mannan 1999). Although this trait may be useful for identifying central species in vertebrate scavenger communities, regardless of the class, the use of class‐specific measures for movement efficiency may constitute a more accurate approach for predicting species centrality, e.g. the hand‐wing index for birds (Sheard et al. 2020).

Activity at night and during crepuscular hours emerged as a key factor promoting species centrality within networks. All species exhibiting this activity pattern were mammals and held central network positions, this finding aligns with prior studies demonstrating the significant role of facultative scavengers in shaping scavenging communities, with the activity patterns of species being a crucial factor (Moleón et al. 2015; Huijbers et al. 2016; Sebastián‐González et al. 2016; Mateo‐Tomás et al. 2017). Three of the observed species—wild boar, marten, and red fox—are meso‐predators, while one—the wolf—functions as an apex predator. Being active at night and in the crepuscule may be an adaptation in response to human disturbances (Gaynor et al. 2018), particularly in the case of the species that are hunted in the study sites, e.g., wild boar, but can also be due to temporal niche partitioning that enables mammals to avoid direct competition with day‐active species that are functionally dominant (i.e., vultures; Mateo‐Tomás et al. 2017; Mateo‐Tomás and Olea 2018; Olea, Iglesias, and Mateo‐Tomás 2022) and may thus increase their own centrality. Nevertheless, shifts from natural patterns of activity due to human disturbance can have detrimental consequences for fitness, population persistence, community interactions, and evolution (Gaynor et al. 2018).

Noisiness promoted species' centrality; noisy species may be indicators of carrion location and so increase the chances of both con‐ and heterospecific scavengers locating a carcass (Dermody, Tanner, and Jackson 2011; Sebastián‐González et al. 2021). The observed noisy species were only birds, mainly corvids and griffon vultures. Previous studies show that flocks of corvids are an unequivocal sign of carrion presence and often attract eagles and other raptors (Selva 2004; Olea, Iglesias, and Mateo‐Tomás 2022). In fact, the activity of vultures closely followed those of ravens and carrion crows in the study site in northern Spain, suggesting the existence of interspecific facilitative interactions (Olea, Iglesias, and Mateo‐Tomás 2022). For instance, the earlier detection of carcasses by corvids could increase carcass detectability by vultures, which in turn, could facilitate corvids to access carrion. This finding confirms the hypothesis that noisy species play an important role in networks by signaling carrion location and potentially promoting turnover in species.

Although several functional traits determined the role of the species in scavenger networks, our results also highlight that such roles can be affected by local conditions. Previous studies indicate that context‐dependent factors (e.g., habitat, species abundance) influence the structure, composition, and functioning of scavenger communities (Mateo‐Tomás et al. 2017; Turner et al. 2017; Pardo‐Barquín, Mateo‐Tomás, and Olea 2019). For instance, scavengers, such as the common raven and the cinereous vulture, assumed both central and peripheral roles at different sites. The common raven assumed centrality in the Cordillera Cantábrica but played a peripheral role in the Mediterranean network of Sierra Morena. Conversely, the cinereous vulture held centrality in the Mediterranean networks but appeared peripheral in the northern regions of Spain. Species abundance strongly influences scavenger community functioning and our findings likely mirror shifts in scavenger species abundance across Spain (Mateo‐Tomás et al. 2017). Notably, the corvid population is smaller in Mediterranean Spain, while the cinereous vulture is vagrant in the northern regions (Del Moral 2003; De la Puente, Moreno‐Opo, and González 2007). Declines in scavenger species abundance can thus alter the role of species and threaten the stability of the ecological processes and services supported by these species.

Species with large home ranges (> 40,000 km^2^) often had central roles in scavenger networks and play a pivotal role in network cohesion. Thus, the spatial scale of conservation measures is vital for these species, and transboundary conservation measures beyond administrative boundaries are imperative. For instance, vertebrate scavenger conservation is strongly related to livestock carcass availability in Europe (e.g., Tella 2001), and the European sanitary regulations allowing the abandonment of livestock carcasses for feeding scavengers are unevenly implemented across the region (Mateo‐Tomás, Olea, and López‐Bao 2018; Mateo‐Tomás, Olea, López‐Bao, et al. 2019; Mateo‐Tomás, Olea, Selva, et al. 2019). Additionally, many vertebrate species with central roles in the scavenger networks, such as vultures, corvids, apex predators, and mesocarnivores are severely affected by poisoning and other forms of illegal wildlife persecution in Spain and elsewhere (Fernández‐García et al. 2024; Ogada et al. 2016). Coordinated, regional conservation policy is needed to protect key scavenger species and the ecosystem functions and services they provide, but actions to combat threats to these species differ not only at international but also at national scales (Cano 2017; López‐Bao and Mateo‐Tomás 2022).

Overall, our analysis of multiple scavenger networks reveals that the functional roles of species within a community can be predicted by functional traits. This information can facilitate the identification of species crucial for the conservation of scavenging functions, and for predicting species vulnerability to ecological changes. In general, scavenger species with large home ranges emerge as pivotal players in shaping ecological networks. These species play a critical role in connecting different networks, and forming and maintaining metacommunities (Mittelbach and Schemske 2015). Therefore, species with large home ranges, like vultures, may impact the structure and functioning of not only their local network but also that of other ecosystems (Mateo‐Tomás, Olea, López‐Bao, et al. 2019; Mateo‐Tomás, Olea, Selva, et al. 2019), although this role would be further mediated by the species abundance. Our findings underscore the need for coordinated cross‐boundary protection efforts for wide‐ranging scavenger species. Integrating functional traits and trophic roles into current management strategies, along with a better understanding of transboundary dynamics, are helpful, if not necessary steps to guarantee the resilience and functionality of scavenger networks.

## Author Contributions

**Violeta Marie Montenegro:** conceptualization (lead), formal analysis (lead), investigation (lead), methodology (lead), visualization (lead), writing – original draft (lead). **Patricia Mateo‐Tomás:** data curation (lead), methodology (supporting), resources (supporting), writing – review and editing (supporting). **Jessica Schneider:** project administration (supporting), visualization (supporting), writing – review and editing (supporting). **Daisy H. Dent:** writing – review and editing (supporting). **Tom Crowther:** writing – review and editing (supporting). **Carolina Bello:** conceptualization (supporting), investigation (supporting), methodology (supporting), supervision (lead), writing – original draft (supporting), writing – review and editing (lead).

## Conflicts of Interest

The authors declare no conflicts of interest.

## Supporting information


Appendix S1.

## Data Availability

The data supporting this study's findings are available for all interested parties on a shared drive accessible with the following link: https://drive.google.com/drive/folders/15GotxzA0dCaRBoywpXEILZMiHrv7OHDY?usp=sharing.

## References

[ece370485-bib-0095] Agencia Estatal de Metereologia (AEMET), Ministerio De Medio Ambiente y Medio Rural y Marino . 2011. “Atlas Climático Ibérico.” Madrid, Spain. 15–18.

[ece370485-bib-0001] Aubin, I. , L. Venier , J. Pearce , and M. Moretti . 2013. “Can a Trait‐Based Multi‐Taxa Approach Improve Our Assessment of Forest Management Impact on Biodiversity?” Biodiversity and Conservation 22: 2957–2975.

[ece370485-bib-0002] Bartomeus, I. , D. Gravel , J. M. Tylianakis , M. A. Aizen , I. A. Dickie , and M. Bernard‐Verdier . 2016. “A Common Framework for Identifying Linkage Rules Across Different Types of Interactions.” Functional Ecology 30: 1894–1903.

[ece370485-bib-0003] Bascompte, J. 2003. “Extinction Thresholds: Insights From Simple Models.” Annales Zoologici Fennici 40: 99–114.

[ece370485-bib-0004] Bascompte, J. 2009. “Disentangling the Web of Life.” Science 325: 416–419. 19628856 10.1126/science.1170749

[ece370485-bib-0005] Billerman, M. , B. K. Keeney , P. G. Rodewald , and T. S. Schulenberg . 2022. Birds of the World. Ithaca: Cornell Lab of Ornithology.

[ece370485-bib-0006] Butler, J. R. A. , and J. T. du Toit . 2002. “Diet of Free‐Ranging Domestic Dogs (Canis Familiaris) in Rural Zimbabwe: Implications for Wild Scavengers on the Periphery of Wildlife Reserves.” Animal Conservation Forum 5: 29–37.

[ece370485-bib-0007] Calcagno, V. , and C. de Mazancourt . 2010. “Glmulti: An R Package for Easy Automated Model Selection With (Generalized) Linear Models.” Journal of Statistical Software 34: 1–29.

[ece370485-bib-0008] Cano, C. 2017. La lucha contra el veneno en España (2011–2016). Madrid: Clasificación por Comunidades Autónomas.

[ece370485-bib-0009] Cirtwill, A. R. , G. V. Dalla Riva , M. P. Gaiarsa , et al. 2018. “A Review of Species Role Concepts in Food Webs.” Food Webs 16: e00093.

[ece370485-bib-0010] Cortés‐Avizanda, A. , G. Blanco , T. L. Devault , et al. 2016. “Supplementary Feeding and Endangered Avian Scavengers: Benefits, Caveats, and Controversies.” Frontiers in Ecology and the Environment 14: 191–199.

[ece370485-bib-0011] Costa, L. D. F. , F. A. Rodrigues , G. Travieso , and P. R. V. Boas . 2007. “Characterization of Complex Networks: A Survey of Measurements.” Advances in Physics 56: 167–242.

[ece370485-bib-0012] Coux, C. , R. Rader , I. Bartomeus , and J. M. Tylianakis . 2016. “Linking Species Functional Roles to Their Network Roles.” Ecology Letters 19: 762–770. 27169359 10.1111/ele.12612

[ece370485-bib-0013] De la Puente, J. , R. Moreno‐Opo , and J. C. González . 2007. El buitre negro en España. Madrid: Censo Nacional. SEO/BirdLife.

[ece370485-bib-0014] Dehling, M. D. , I. Bender , P. G. Blendinger , et al. 2021. “Specialists and Generalists Fulfil Important and Complementary Functional Roles in Ecological Processes.” Functional Ecology 35: 1810–1821.

[ece370485-bib-0015] Boletín Oficial Del Estado . 2011. “Real Decreto 139/2011, de 4 de febrero, para el desarrollo del Listado de Especies Silvestres en Régimen de Protección Especial y del Catálogo Español de Especies Amenazadas.” BOE 46: 20912–20951.

[ece370485-bib-0016] Del Moral, J. C. 2003. “Atlas de las aves Reproductoras de España. Dirección General de Conservación de la Naturaleza.”

[ece370485-bib-0017] Del Moral, J. C. 2009. El alimoche Comun en España. Poblacion en 2008 y metodo de censo. Madrid: SEO/BirdLife.

[ece370485-bib-0018] Del Moral, J. C. , and B. Molina . 2009. El buitre leonado en España. Población Reproductora en 2008 y método de censo. Madrid: SEO/BirdLife.

[ece370485-bib-0019] Dermody, B. J. , C. J. Tanner , and A. L. Jackson . 2011. “The Evolutionary Pathway to Obligate Scavenging in Gyps Vultures.” PLoS One 6: e24635. 21931786 10.1371/journal.pone.0024635 PMC3169611

[ece370485-bib-0020] DeVault, T. L. , J. Beasley , Z. H. Olson , et al. 2016. “Ecosystem Services Provided by Avian Scavengers.” In Why Birds Matter: Avian Ecological Function and Ecosystem Services, edited by C. H. Şekercioğlu , D. G. Wenny , and C. J. Whelan , vol. 2016, 235–270. Chicago, IL: University of Chicago Press.

[ece370485-bib-0021] DeVault, T. L. , E. Rhodes Olin , and J. A. Shivik . 2003. “Scavenging by Vertebrates: Behavioral, Ecological, and Evolutionary Perspectives on an Important Energy Transfer Pathway in Terrestrial Ecosystems.” Oikos 102: 225–234.

[ece370485-bib-0022] Dormann, C. F. , J. Fründ , N. Blüthgen , and B. Gruber . 2009. “Indices, Graphs and Null Models: Analyzing Bipartite Ecological Networks.” Open Journal of Ecology 2: 7–24.

[ece370485-bib-0023] Ducatez, S. , R. Tingley , and R. Shine . 2014. “Using Species Co‐Occurrence Patterns to Quantify Relative Habitat Breadth in Terrestrial Vertebrates.” Ecosphere 5: art152.

[ece370485-bib-0024] Estrada, E. , and Ö. Bodin . 2008. “Using Network Centrality Measures to Manage Landscape Connectivity.” Ecological Applications 18: 1810–1825. 18839774 10.1890/07-1419.1

[ece370485-bib-0026] Fernández, F. J. 1998. “Buitres Leonados (*Gyps fulvus*) Comiendo Plantas.” El Escribano 17: 2–6.

[ece370485-bib-0027] Fernández‐García, M. , J. V. López‐Bao , P. P. Olea , et al. 2024. “Strengths and Limitations of Official Sources of Wildlife Poisoning.” Biological Conservation 294: 110636.

[ece370485-bib-0028] Gaynor, K. M. , C. E. Hojnowski , N. H. Carter , and J. S. Brashares . 2018. “The Influence of Human Disturbance on Wildlife Nocturnality.” Science 360: 1232–1235. 29903973 10.1126/science.aar7121

[ece370485-bib-0029] Grigg, N. P. , J. M. Krilow , C. Gutierrez‐Ibanez , D. R. Wylie , G. R. Graves , and A. N. Iwaniuk . 2017. “Anatomical Evidence for Scent Guided Foraging in the Turkey Vulture.” Scientific Reports 7: 17408. 29234134 10.1038/s41598-017-17794-0 PMC5727128

[ece370485-bib-0030] Gutiérrez‐Cánovas, C. , M. Moleón , P. Mateo‐Tomás , P. P. Olea , E. Sebastián‐González , and J. A. Sánchez‐Zapata . 2020. “Large Home Range Scavengers Support Higher Rates of Carcass Removal.” Functional Ecology 34: 1921–1932.

[ece370485-bib-0031] Hooper, D. U. , F. S. Chapin III , J. J. Ewel , et al. 2005. “Effects of Biodiversity on Ecosystem Functioning: A Consensus of Current Knowledge.” Ecological Monographs 75: 3–35.

[ece370485-bib-0032] Houston, D. 2001. Vultures & Condors. Scotland: Colin Baxter Photography Ltd.

[ece370485-bib-0033] Huijbers, C. M. , T. A. Schlacher , R. R. McVeigh , et al. 2016. “Functional Replacement Across Species Pools of Vertebrate Scavengers Separated at a Continental Scale Maintains an Ecosystem Function.” Functional Ecology 30: 998–1005.

[ece370485-bib-0034] Inger, R. , D. T. C. Cox , E. Per , B. A. Norton , and K. J. Gaston . 2016. “Ecological Role of Vertebrate Scavengers in Urban Ecosystems in the UK.” Ecology and Evolution 6: 7015–7023. 28725378 10.1002/ece3.2414 PMC5513233

[ece370485-bib-0035] IUCN . 2021. “The IUCN Red List of Threatened Species. Version 2021.” https://www.iucnredlist.org.

[ece370485-bib-0036] IUCN Standards and Petitions Committee . 2022. “Guidelines for Using the IUCN Red List Categories and Criteria.”

[ece370485-bib-0037] Ives, A. M. , M. Brenn‐White , J. Y. Buckley , C. J. Kendall , S. Wilton , and S. L. Deem . 2022. “A Global Review of Causes of Morbidity and Mortality in Free‐Living Vultures.” EcoHealth 19: 40–54. 35000042 10.1007/s10393-021-01573-5

[ece370485-bib-0038] Jones, K. E. , J. Bielby , M. Cardillo , et al. 2009. “PanTHERIA: A Species‐Level Database of Life History, Ecology, and Geography of Extant and Recently Extinct Mammals: Ecological Archives E090‐184.” Ecology 90: 2648.

[ece370485-bib-0039] Jordán, F. , W. Liu , and A. J. Davis . 2006. “Topological Keystone Species: Measures of Positional Importance in Food Webs.” Oikos 112: 535–546.

[ece370485-bib-0040] Kane, A. , K. Healy , T. Guillerme , G. D. Ruxton , and A. L. Jackson . 2017. “A Recipe for Scavenging in Vertebrates–the Natural History of a Behaviour.” Ecography 40: 324–334.

[ece370485-bib-0041] Kane, A. , A. L. Jackson , D. L. Ogada , A. Monadjem , and L. McNally . 2014. “Vultures Acquire Information on Carcass Location From Scavenging Eagles.” Proceedings of the Royal Society B: Biological Sciences 281: 20141072. 10.1098/rspb.2014.1072 PMC4173674 25209935

[ece370485-bib-0042] Laigle, I. , I. Aubin , C. Digel , U. Brose , I. Boulangeat , and D. Gravel . 2018. “Species Traits as Drivers of Food Web Structure.” Oikos 127: 316–326.

[ece370485-bib-0043] Laurindo, R. d. S. , J. Vizentin‐Bugoni , D. C. Tavares , M. C. S. Mancini , R. d. M. Mello , and R. Gregorin . 2020. “Drivers of Bat Roles in Neotropical Seed Dispersal Networks: Abundance Is More Important Than Functional Traits.” Oecologia 193: 189–198. 32405932 10.1007/s00442-020-04662-4

[ece370485-bib-0044] López‐Bao, J. V. , and P. Mateo‐Tomás . 2022. “Wipe Out Highly Hazardous Pesticides to Deter Wildlife Poisoning: The Case of Carbofuran and Aldicarb.” Biological Conservation 275: 109747.

[ece370485-bib-0045] Markandya, A. , T. Taylor , A. Longo , M. N. Murty , S. Murty , and K. Dhavala . 2008. “Counting the Cost of Vulture Decline: An Appraisal of the Human Health and Other Benefits of Vultures in India.” Ecological Economics 67: 194–204.

[ece370485-bib-0046] Mateo‐Tomás, P. , and P. P. Olea . 2018. “Griffon Vultures Scavenging at Night: Trophic Niche Expansion to Reduce Intraspecific Competition?” Ecology 99: 1897–1899. 29889300 10.1002/ecy.2366

[ece370485-bib-0047] Mateo‐Tomás, P. , P. P. Olea , and J. V. López‐Bao . 2018. “Europe's Uneven Laws Threaten Scavengers.” Science 360: 612–613. 10.1126/science.aat8492 29748276

[ece370485-bib-0048] Mateo‐Tomás, P. , P. P. Olea , J. V. López‐Bao , P. González‐Quirós , and P. Peón . 2019. “Different Criteria for Implementing Sanitary Regulations Lead to Disparate Outcomes for Scavenger Conservation.” Journal of Applied Ecology 56: 500–508.

[ece370485-bib-0049] Mateo‐Tomás, P. , P. P. Olea , M. Moleón , N. Selva , and J. A. Sánchez‐Zapata . 2017. “Both Rare and Common Species Support Ecosystem Services in Scavenger Communities.” Global Ecology and Biogeography 26: 1459–1470.

[ece370485-bib-0050] Mateo‐Tomás, P. , P. P. Olea , M. Moleón , et al. 2015. “From Regional to Global Patterns in Vertebrate Scavenger Communities Subsidized by Big Game Hunting.” Diversity and Distributions 21: 913–924.

[ece370485-bib-0051] Mateo‐Tomás, P. , P. P. Olea , N. Selva , and J. A. Sánchez‐Zapata . 2019. “Species and Individual Replacements Contribute More Than Nestedness to Shape Vertebrate Scavenger Metacommunities.” Ecography 42: 365–375.

[ece370485-bib-0052] McGill, B. J. , B. J. Enquist , E. Weiher , and M. Westoby . 2006. “Rebuilding Community Ecology From Functional Traits.” Trends in Ecology & Evolution 21: 178–185. 16701083 10.1016/j.tree.2006.02.002

[ece370485-bib-0053] Medeiros, L. P. , G. Garcia , J. N. Thompson , and P. R. Guimaraes Jr. 2018. “The Geographic Mosaic of Coevolution in Mutualistic Networks.” Proceedings of the National Academy of Sciences 115: 12017–12022. 10.1073/pnas.1809088115 PMC6255164 30404910

[ece370485-bib-0054] Meretsky, V. J. , and R. W. Mannan . 1999. “Supplemental Feeding Regimes for Egyptian Vultures in the Negev Desert, Israel.” Journal of Wildlife Management 63: 107–115.

[ece370485-bib-0055] Mittelbach, G. G. , and D. W. Schemske . 2015. “Ecological and Evolutionary Perspectives on Community Assembly.” Trends in Ecology & Evolution 30: 241–247. 25804867 10.1016/j.tree.2015.02.008

[ece370485-bib-0056] Moleón, M. , J. A. Sánchez‐Zapata , E. Sebastián‐González , and N. Owen‐Smith . 2015. “Carcass Size Shapes the Structure and Functioning of an African Scavenging Assemblage.” Oikos 124: 1391–1403.

[ece370485-bib-0057] Moleón, M. , J. A. Sánchez‐Zapata , N. Selva , J. A. Donázar , and N. Owen‐Smith . 2014. “Inter‐Specific Interactions Linking Predation and Scavenging in Terrestrial Vertebrate Assemblages.” Biological Reviews 89: 1042–1054. 24602047 10.1111/brv.12097

[ece370485-bib-0058] Morales‐Castilla, I. , M. G. Matias , D. Gravel , and M. B. Araújo . 2015. “Inferring Biotic Interactions From Proxies.” Trends in Ecology & Evolution 30: 347–356. 25922148 10.1016/j.tree.2015.03.014

[ece370485-bib-0059] Morales‐Reyes, Z. , J. M. Pérez‐García , M. Moleón , et al. 2016. “Evaluation of the Network of Protection Areas for the Feeding of Scavengers in Spain: From Biodiversity Conservation to Greenhouse Gas Emission Savings.” Journal of Applied Ecology 54: 1120–1129.

[ece370485-bib-0060] Morales‐Reyes, Z. , J. M. Pérez‐García , M. Moleón , et al. 2015. “Supplanting Ecosystem Services Provided by Scavengers Raises Greenhouse Gas Emissions.” Scientific Reports 5: 7811. 25589381 10.1038/srep07811 PMC4295086

[ece370485-bib-0061] Moreno‐Opo, R. , and A. Margalida . 2019. “Human‐Mediated Carrion: Effects on Ecological Processes.” In Carrion Ecology and Management. Wildlife Research Monographs, vol 2, edited by P. Olea , P. Mateo‐Tomás , and J. Sánchez‐Zapata , 183–211. Cham: Springer.

[ece370485-bib-0062] Moreno‐Opo, R. , A. Trujillano , and A. Margalida . 2020. “Larger Size and Older Age Confer Competitive Advantage: Dominance Hierarchy Within European Vulture Guild.” Scientific Reports 10: 2430. 32051486 10.1038/s41598-020-59387-4 PMC7015885

[ece370485-bib-0063] Moulatlet, G. M. , W. Dáttilo , and F. Villalobos . 2023. “Species‐Level Drivers of Avian Centrality Within Seed‐Dispersal Networks Across Different Levels of Organisation.” Journal of Animal Ecology 92: 2126–2137. 37454385 10.1111/1365-2656.13986

[ece370485-bib-0064] Myers, P. , R. Espinosa , C. S. Parr , T. Jones , G. S. Hammond , and T. A. Dewey . 2006. “The Animal Diversity Web.” http://animaldiversity.org.

[ece370485-bib-0065] Myhrvold, N. P. , E. Baldridge , B. Chan , D. Sivam , D. L. Freeman , and S. K. M. Ernest . 2015. “An Amniote Life‐History Database to Perform Comparative Analyses With Birds, Mammals, and Reptiles: Ecological Archives E096‐269.” Ecology 96: 3109.

[ece370485-bib-0066] Namba, T. , K. Tanabe , and N. Maeda . 2008. “Omnivory and Stability of Food Webs.” Ecological Complexity 5: 73–85.

[ece370485-bib-0067] Oaks, J. L. , M. Gilbert , M. Z. Virani , et al. 2004. “Diclofenac Residues as the Cause of Vulture Population Decline in Pakistan.” Nature 427: 630–633. 14745453 10.1038/nature02317

[ece370485-bib-0068] O'Bryan, C. J. , A. R. Braczkowski , H. L. Beyer , N. H. Carter , J. E. M. Watson , and E. McDonald‐Madden . 2018. “The Contribution of Predators and Scavengers to Human Well‐Being.” Nature Ecology & Evolution 2: 229–236. 29348647 10.1038/s41559-017-0421-2

[ece370485-bib-0069] O'Bryan, C. J. , M. H. Holden , and J. E. M. Watson . 2019. “The Mesoscavenger Release Hypothesis and Implications for Ecosystem and Human Well‐Being.” Ecology Letters 22: 1340–1348. 31131976 10.1111/ele.13288

[ece370485-bib-0070] Ogada, D. , P. Shaw , R. L. Beyers , et al. 2016. “Another Continental Vulture Crisis: Africa's Vultures Collapsing Toward Extinction.” Conservation Letters 9: 89–97.

[ece370485-bib-0071] Olea, P. P. , N. Iglesias , and P. Mateo‐Tomás . 2022. “Temporal Resource Partitioning Mediates Vertebrate Coexistence at Carcasses: The Role of Competitive and Facilitative Interactions.” Basic and Applied Ecology 60: 63–75.

[ece370485-bib-0072] Pardo‐Barquín, E. , P. Mateo‐Tomás , and P. P. Olea . 2019. “Habitat Characteristics From Local to Landscape Scales Combine to Shape Vertebrate Scavenging Communities.” Basic and Applied Ecology 34: 126–139.

[ece370485-bib-0073] Pontzer, H. 2007. “Effective Limb Length and the Scaling of Locomotor Cost in Terrestrial Animals.” Journal of Experimental Biology 210: 1752–1761. 17488938 10.1242/jeb.002246

[ece370485-bib-0074] Prakash, V. , D. J. Pain , A. A. Cunningham , et al. 2003. “Catastrophic Collapse of Indian White‐Backed Gyps Bengalensis and Long‐Billed Gyps Indicus Vulture Populations.” Biological Conservation 109: 381–390.

[ece370485-bib-0075] Prugh, L. R. , C. J. Stoner , C. W. Epps , et al. 2009. “The Rise of the Mesopredator.” Bioscience 59: 779–791.

[ece370485-bib-0076] RStudio Team . 2022. RStudio: Integrated Development for R. Boston, MA: RStudio, PBC. http://www.rstudio.com/.

[ece370485-bib-0077] Ruxton, G. D. , and D. C. Houston . 2004. “Obligate vertebrate scavengers must be large soaring fliers.” Journal of Theoretical Biology 228: 431–436. 15135041 10.1016/j.jtbi.2004.02.005

[ece370485-bib-0078] Schlichting, P. E. , C. N. Love , S. C. Webster , and J. C. Beasley . 2019. “Efficiency and Composition of Vertebrate Scavengers at the Land‐Water Interface in the Chernobyl Exclusion Zone.” Food Webs 18: e00107.

[ece370485-bib-0079] Sebastián‐González, E. , M. Moleón , J. P. Gibert , et al. 2016. “Nested Species‐Rich Networks of Scavenging Vertebrates Support High Levels of Interspecific Competition.” Ecology 97: 95–105. 27008779 10.1890/15-0212.1

[ece370485-bib-0080] Sebastián‐González, E. , Z. Morales‐Reyes , F. Botella , et al. 2021. “Functional Traits Driving Species Role in the Structure of Terrestrial Vertebrate Scavenger Networks.” Ecology 102: e03519. 34449876 10.1002/ecy.3519

[ece370485-bib-0081] Sebastián‐González, E. , Z. Morales‐Reyes , F. Botella , et al. 2020. “Network Structure of Vertebrate Scavenger Assemblages at the Global Scale: Drivers and Ecosystem Functioning Implications.” Ecography 43: 1143–1155.

[ece370485-bib-0082] Selva, N. 2004. “The Role of Scavenging in the Predator Community of Bialowieza Primeval Forest.” PhD thesis, University of Sevilla.

[ece370485-bib-0083] Selva, N. , and M. A. Fortuna . 2007. “The Nested Structure of a Scavenger Community.” Proceedings of the Royal Society B: Biological Sciences 274: 1101–1108. 10.1098/rspb.2006.0232 PMC2124470 17301021

[ece370485-bib-0084] Selva, N. , B. Jedrzejewska , W. Jedrzejewski , and A. Wajrak . 2003. “Scavenging on European Bison Carcasses in Bialowieza Primeval Forest (Eastern Poland).” Ecoscience 10: 303–311.

[ece370485-bib-0085] Selva, N. , M. Moleón , E. Sebastián‐González , et al. 2019. “Vertebrate Scavenging Communities.” Carrion Ecology and Management. Wildlife Research Monographs 2: 71–99.

[ece370485-bib-0086] Sheard, C. , M. H. C. Neate‐Clegg , N. Alioravainen , et al. 2020. “Ecological Drivers of Global Gradients in Avian Dispersal Inferred From Wing Morphology.” Nature Communications 11: 2463. 10.1038/s41467-020-16313-6 PMC7235233 32424113

[ece370485-bib-0088] Tella, J. L. 2001. “Action Is Needed Now, or BSE Crisis Could Wipe out Endangered Birds of Prey.” Nature 410: 408. 10.1038/35068717 11260682

[ece370485-bib-0089] Tobias, J. A. , C. Sheard , A. L. Pigot , et al. 2022. “AVONET: Morphological, Ecological and Geographical Data for all Birds.” Ecology Letters 25: 581–597. 35199922 10.1111/ele.13898

[ece370485-bib-0090] Turner, K. L. , E. F. Abernethy , L. M. Conner , O. E. Rhodes , and J. C. Beasley . 2017. “Abiotic and Biotic Factors Modulate Carrion Fate and Vertebrate Scavenging Communities.” Ecology 98: 2413–2424. 28628191 10.1002/ecy.1930

[ece370485-bib-0091] Wilman, H. , J. Belmaker , J. Simpson , C. de la Rosa , M. M. Rivadeneira , and W. Jetz . 2014. “EltonTraits 1.0: Species‐Level Foraging Attributes of the world's Birds and Mammals: Ecological Archives E095‐178.” Ecology 95: 2027.

[ece370485-bib-0092] Wilson, E. E. , and E. M. Wolkovich . 2011. “Scavenging: How Carnivores and Carrion Structure Communities.” Trends in Ecology & Evolution 26: 129–135. 21295371 10.1016/j.tree.2010.12.011

[ece370485-bib-0093] Yamaç, E. , and E. Günyel . 2010. “Diet of the Eurasian Black Vulture, Aegypius Monachus Linnaeus, 1766, in Turkey and Implications for Its Conservation.” Zoology in the Middle East 51: 15–22.

[ece370485-bib-0094] Zepeda Mendoza, M. L. , M. Roggenbuck , K. Manzano Vargas , et al. 2018. “Protective Role of the Vulture Facial Skin and Gut Microbiomes Aid Adaptation to Scavenging.” Acta Veterinaria Scandinavica 60: 61. 30309375 10.1186/s13028-018-0415-3 PMC6182802

