# Supplementary material for: Linking Functional Traits To Trophic Roles In Scavenger Assemblages
Source: Ecol Evol. 2025 Jan 8;15(1):e70485. doi: 10.1002/ece3.70485 (PMC11711106; doi:10.1002/ece3.70485)
Supplement: Supplementary file 1 — Appendix S1. [file ECE3-15-e70485-s001.docx]

# LINKING FUNCTIONAL TRAITS TO TROPHIC ROLES IN SCAVENGER ASSEMBLAGES

## Supplementary materials

Supplementary material 1 – Detailed methods

Supplementary material 2 – Additional graphs and results

### Supplementary material 1

#### Methods

Based on the camera records, we created a carcass-scavenger species interaction network for each location. Each scavenger network is a quantitative adjacent matrix where row *i* represents a carcass species (lower trophic level) and column *j* a scavenger species (higher trophic level). The matrix cells *a_ij_* were filled with the number of times the interaction between a scavenger species and a carcass species was recorded (i.e. the '*strength*' of the interaction between those two species). The networks built by the feeding interactions between scavenger and carcass species constitute bipartite networks, where species are separated into two groups (scavengers and carcasses) that interact with each other, but not within themselves (Costa et al., 2007). Hence, a scavenger can't interact with another scavenger except through carrion.

##### Scavenger species' life-history traits

We compiled 23 species' life-history traits that can influence the trophic role of the scavenger species, including physical traits such as size, as well as network traits such as trophic, social, or mobility characteristics (Table S2). Additionally, we looked at species conservation characteristics that are typically related to species size (Gaston & Blackburn, 1997). Scavenger functional traits were obtained from the databases AVONET (Tobias et al., 2022), EltonTraits (Wilman et al., 2014), Amniota (Myhrvold et al., 2015), and PanTHERIA (Jones et al., 2009), as well as the online sources Animal Diversity Web (Myers et al., 2006) and Birds of the World (Billerman et al., 2022). We also considered the scavenger species conservation status at national level (RD 139/2011; Boletín Oficial Del Estado, 2011), which is related with species’ abundance and distribution (IUCN Standards and Petitions Committee, 2022) and can therefore explain their role in the networks. For observations that were not determined to genus rather than species level, we imputed the trait information by using the mean from the genus-level information. For *Martes spp*. we used the mean values of *Martes foina* and *Martes martes*. We examined the correlation and collinearity among functional traits recorded as continuous variables by using a pairwise Pearson correlation and running a Variation Inflation Factor (VIF) analysis on R (version 4.3.1; RStudio Team, 2022). We selected the continuous variables that exhibited only weak or no correlation with each other (r_P <_ |0.70|) (Figure S1). For the analysis, we selected a set of 13 morphological, life-history and functional traits that had information for at least 70% of the species and did not correlate with each other (Table 1).

##### The trophic role of scavengers

To measure the trophic role of the scavengers, we used species-level metrics that characterize the species' position in the network (Cirtwill et al., 2018). We calculated four metrics using the 'bipartite package' (version 2.18) in R (Dormann et al., 2009): (i) normalized degree (ND) (ii) specialization (d'), (iii) closeness centrality (C_c_) and (iv) betweenness centrality (C_b_). These metrics describe whether a species is central or peripheral in the network. The normalized degree is the sum of the realized interactions of a species (i.e., *k_i_*), divided by the number of carrion species censused at the network (*k_max_*), thus constituting a local metric that describes species k_i_'s direct interactions:

$ND=\frac{k}{k_{max}}$ (1)

Species with high degree are important because they interact with most carrion species, and are fundamental to the network structure. Additionally, species that significantly influence the structure of scavenger networks could have a particularly significant impact on scavenging efficiency (i.e. carrion consumption rate), regardless of the amount of carrion they consume. For instance, certain scavengers can locate and access most carcasses, potentially exerting a greater influence on the composition of their community and the control of scavenging activities (Sebastián‐González et al., 2021). In this context, degree serves as an indicator of a species' importance in both the structure and functioning of its community.

The d' metric (ii) describes the level of specialization of a scavenger species using carrion based on its discrimination from a random selection of the resource (Dormann et al., 2009). It ranges from 0 (not specialist) to 1 (specialist) (Blüthgen et al., 2006). Generalist species interact with many carrion species that are abundant, whereas specialists are those that use few and rare resources. Specialist species are more vulnerable to environmental perturbations, such as species extinctions in their communities or variations in carrion availability (Allesina & Tang, 2012; Cirtwill et al., 2018). The d' metric takes the number of interactions as a proxy for the abundance of carrion, constituting a quantitative measure. Then, d' is the normalized version of the Kullback Leibler distance (d)

$d_{i}=\sum_{j=1}^{c} {p'}_{ij}\cdot\ln\frac{{p'}_{ij}}{qj}$, (2.1)

which compares the distribution of the interactions of a species with each partner p'_ij_ to the overall partner availability q_j_

${p'}_{ij}=a_{ij}/A_{i}$ (2.2)

with a_ij_ being the number of interactions between a scavenger and a carcass species, and A_i_ the total number of scavenger species that visit a carrion species. And q_j_ is the proportion of all visits to carcass *j* concerning the total number of interactions in the network *m.* Thus, it describes the availability of that of carcasses from a carrion species in comparison to species providing carcasses in the study area:

$q_{j}={A_{J}}/m$ (2.3)

The normalized d (i.e., d'), is based on the theoretical maximum of d given by d_max_ = ln (m/A_i_) and the theoretical minimum (d_min_), which is zero for the special case where all p'_ij_ = q*_j_*. However, a more realistic d_min_ for each network is computed by the algorithm of the bipartite network (see Blüthgen et al., 2006).

${d'}_{i}= \frac{d_{i}-d_{min}}{d_{max}-d_{min}}$ (2.4)

Closeness centrality (iii) describes how distant a scavenger is to all other species in the community. As such, it reflects the position within the global structure of the network. Specifically, it indicates how rapidly or efficiently a species is likely to influence the overall community if, for example, there is a perturbation, such as the loss of that species (Estrada & Bodin, 2008). Closeness centrality is calculated as the inverse of the average shortest distance from the focal node to each node in the network.

$C_{c}=\left( \sum_{j=1, j\neq k}^{n} \frac{d_{jk}}{n-1} \right)^{-1}$ (3)

where d is the distance between species *j* and focal species *k*, and n is the number of species in the network. Peripheral species have low closeness centrality scores, while central species have high closeness centrality.

Betweenness centrality (iv) describes the number of times a species finds itself on the shortest ‘path’ between two species, with the path consisting of species that connect the pair of species in question, which do not interact with each other directly. Therefore, betweenness centrality identifies species that act as a ‘bridge’ for energy transfer within the network (Cirtwill et al., 2018), which if removed, would disconnect the network into multiple components. Species with high betweenness are thus important to maintain connectivity in a network. For instance, a scavenger feeding on two different carcass species, which are otherwise eaten by species specialists, is important for keeping these two network components together. Betweenness centrality is defined as

$C_{b}=2 \sum_{i<j, k\neq i} \frac{{g_{ij}(k)}/{g_{ij}}}{\left( n-1 \right)(n-2)}$ (4)

Where n is the number of species in a network, g_ij_ is the total number of shortest paths between two species i and j, and g_ij_ (k) is the number of shortest paths between the two species i and j that go through species k.

The four metrics described here were combined using a principal component analysis (PCA) to obtain a single centrality metric summarizing the information on the trophic role of scavenger species at each site (Medeiros et al., 2018; Moulatlet et al., 2023). PC1 negatively correlated with the metrics which indicate higher centrality (i.e., normalized degree, closeness centrality, and betweenness centrality) and positively correlated with specialization d', which indicates lower centrality. To facilitate the interpretation and ranking of species based on their centrality, species’ variable loadings and PC scores were transformed by multiplying them by -1 (i.e., ‘centrality score’ hereafter). Since metric values were computed separately for each species in each network, those species present in multiple networks had more than one centrality score.

##### Data analysis

To explore the relationship between functional traits and the trophic role of vertebrate scavengers in the networks, we fitted Generalized Linear Mixed Models (GLMM) and performed a model selection analysis by exhaustive screening. Species centrality scores for each network was modelled as the response variable using a Gaussian distribution. In the first step, we selected the optimal structure of the random component using REML estimators to compare models with different random structures. We included all selected predictor variables and one of the following random intercepts: network ID, class (i.e., Aves, Mammalia) or their combination (i.e., network ID and class). We fitted random intercept (e.g., 1 | class) and random slope models (RIRS) with the different continuous variables (e.g., body size | class). We used Akaike's information criterion (AIC) to evaluate which models had the best random structure. Models with a difference of less than two values of AIC difference were considered equally good. The models with random intercepts ‘network ID’, ‘class’ and both ‘network ID and class’ and without random slope were equally good (i.e., 𝞓AIC<2, Table S3) and showed a consistent trend in the parameter relationships (Tables S4). Therefore, both ‘network ID’ and ‘class’ were used for the analysis as random components.

In the next step, we searched for the optimal fixed structure of the model. For this, we adopted a multi-model inference approach using R package ‘glmulti’ (version 1.0.8; Calcagno & de Mazancourt, 2010). The function ‘glmulti’ fits all possible combinations and subsets of the predictor variables (i.e., scavengers’ functional traits in our case) for a specified model (we used linear mixed-effects models). We used ‘glmulti’ to fit LMMs for species centrality score as the response variable with all the combinations of functional traits together with the random components ‘network ID’ and ‘class’. We ranked the generated models using Akaike’s information criterion corrected for small sample sizes (AICc). We selected all the best models (i.e., the model with the lowest AICc and those differing less than 2 AICc units from it), and implemented the model-averaging function ‘coef’ from the same package to obtain averaged parameter estimates. We identified the most important variables using their relative evidence weight, computed as the sum of all the evidence weights of all models in which the term appears (Calcagno & de Mazancourt, 2010).

A two-sample Wilcoxon rank sum test was also performed to evaluate whether the difference between obligate and facultative scavenger performance was significant regarding their centrality score. Additionally, we used a Kruskal-Wallis test to explore the differences in centrality among species with different conservation statuses. All data analysis was performed in R version 4.3.1 (RStudio Team, 2022).

### Supplementary material 2

**Table S1.** Species appearing in the three networks. Species in bold are present in all ecosystems. *Martes spp. consists of Martes foina and Martes martes. For the analysiswe used the mean of the traits these species. ** Milvus migrans and Neophron percnopterus are migratory species. Distribution information was summarised from the Atlas of Reproductive Birds of Spain (Marti & del Moral, 2003).

| **Class** | **Cordillera Cantábrica** | **Montes de Toledo** | **Sierra Morena** | **Distribution** |
| --- | --- | --- | --- | --- |
| Bird | *--* | *Accipiter gentilis* | *--* | All across Spain; more dense in the north. |
| Bird | ***Aegypius monachus*** | ***Aegypius monachus*** | ***Aegypius monachus*** | South and west of Spain. |
| Mammal | *Apodemus sp.* | *Apodemus sp.* |  |  |
| Bird | *--* | *Aquila adalberti* | *Aquila adalberti* | Central and southern Spain |
| Bird | ***Aquila chrysaetos*** | ***Aquila chrysaetos*** | ***Aquila chrysaetos*** |  |
| Bird | *--* | *--* | *Bubo bubo* |  |
| Bird |  |  | *Aquila fasciata* |  |
| Bird |  |  | *Bubo bubo* |  |
| Bird | *Buteo buteo* | *Buteo buteo* | *--* |  |
| Mammal | ***Canis familiaris*** | ***Canis familiaris*** | ***Canis familiaris*** |  |
| Mammal | *Canis lupus* | *--* | *--* | Northern Spain, small population Sierra Morena |
| Bird | *--* | *Circus aeruginosus* | *--* |  |
| Bird | ***Corvus corax*** | ***Corvus corax*** | ***Corvus corax*** |  |
| Bird | *Corvus corone* | *--* | *--* |  |
| Bird | *--* | *Cyanopica cyanus* | *Cyanopica cyanus* |  |
| Mammal |  |  | *Felis catus* |  |
| Bird | *--* | *Garrulus glandarius* | *Garrulus glandarius* |  |
| Mammal | ***Genetta genetta*** | ***Genetta genetta*** | ***Genetta genetta*** |  |
| Bird | ***Gyps fulvus*** | ***Gyps fulvus*** | ***Gyps fulvus*** | Sierra Morena has higher abundance than the other sites. |
| Mammal | *--* | *--* | *Herpestes ichneumon* |  |
| Mammal | ***Martes spp.*** | ***Martes foina*** | ***Martes foina*** |  |
| Bird | *--* | *Milvus migrans*** | *--* |  |
| Bird | *Milvus milvus* | *Milvus milvus* | *--* |  |
| Bird | *Neophron percnopterus*** | *Neophron percnopterus*** | *--* | Mostly in northern Spain. Other populations in and central-western and southern Spain. |
| Bird | ***Pica pica*** | ***Pica pica*** | ***Pica pica*** |  |
| Mammal | ***Sus scrofa*** | ***Sus scrofa*** | ***Sus scrofa*** |  |
| Mammal | *Ursus arctos* | *--* | *--* |  |
| Mammal | ***Vulpes vulpes*** | ***Vulpes vulpes*** | ***Vulpes vulpes*** |  |
| **N. mammals** | 9 | 6 | 7 |  |
| **N. birds** | 8 | 15 | 11 |  |

**Table S2.** Functional traits before selection, with their description and completeness.

| **Variable name** | **Variable Definition** | **Units** | **Variable Type** | **Completeness** |
| --- | --- | --- | --- | --- |
| Sociality | Foraging behaviour; describes whether a species forages alone or in groups. Species were defined as “social” if they forage in groups or with family, and “solitary”, if they forage alone. | - | Categorical | 1 |
| Mobility | Mobility capacity. Species were defined as “aerial” if they could fly and “terrestrial” if they moved on ground. | . | Categorical | 1 |
| Scavenging behaviour | Scavenging specialisation. Obligate scavenger species were defined as “obligate”; scavengers that don’t rely on carcasses were defined as “facultative” . | - | Categorical | 1 |
| Predation | Predation behaviour. Species were defined as “non-predator”, “meso-predator” and “top-predator”. Top predators are defined as large mammals at the top of food webs. Meso predators were medium-sized meat-eating species; they can kill their prey. Non-predators were vultures and small generalist species (i.e. *Apodemus spp*.) | - | Categorical | 1 |
| Activity | Activity cycle of each species. Nocturnal: species activity concentrates at night, from sunset to sunrise; diurnal: species activity concentrates during the daylight, from sunrise to sunset; both: the species can be active during night and day. | - | Categorical | 1 |
| Sight | Eyesight capacity. Can be “high” of “low”. |  | Categorical | 0.9 |
| Olfaction | Olfactory capacity. Can be “high” of “low”. |  | Categorical | 1 |
| Noisiness | Use of vocalisations during foraging. As  “Noisy” was a defined species that vocalizes loudly while foraging.  “Quiet” is a species that forages quietly. |  | Categorical | 0.93 |
| Habitat breadth | Index of habitat specialization based on patterns of species co-occurrence calculated by Ducatez et al. 2014. A generalist species occur in a range of habitat categories that vary considerably in species composition, whereas a specialist species is found only in habitats that contain a consistent suite of other species. Habitat breadth for a given species was measured as b 1⁄4 c/l(a), where c is the cumulative number of different species that occur in the habitats used by the species considered and l(a) is the mean habitat species richness calculated over the different habitats used by that species. I used the global multiplicative habitat breadth index. It varies from 1 to more than 7 (Ducatez et al. 2014). | - | Continuous | 0.93 |
| Beak length culmen | AVONET “Length from the tip of the beak to the base of the skull” | mm | Continuous | 0.6 |
| Tarsus length | Length of the tarsus from the posterior notch between tibia and tarsus, to the end of the last scale of acrotarsium (at the bend of the foot) | mm | Continuous | 0.6 |
| Kipps distance | AVONET: “Length from the tip of the first secondary feather to the tip of the longest primary.” | mm | Continuous | 0.6 |
| Tail length | Length of the tail.  AVONET “Distance between the tip of the longest rectrix and the point at which the two central rectrices protrude from the skin, typically measured using a ruler inserted between the two central rectrices [mm].” | mm | Continuous | 1 |
| Trophic niche | Frugivore = species obtaining at least 60% of food resources from fruit;  Granivore = species obtaining at least 60% of food resources from seeds or nuts;  Nectarivore = species obtaining at least 60% of food resources from nectar;  Herbivore = species obtaining at least 60% of food resources from other plant materials in non-aquatic systems, including leaves, buds, whole flowers etc.;  Herbivore aquatic = species obtaining at least 60% of food resources from plant materials in aquatic systems, including algae and aquatic plant leaves;  Invertivore = species obtaining at least 60% of food resources from invertebrates in terrestrial systems, including insects, worms, arachnids, etc.;  Vertivore = species obtaining at least 60% of food resources from vertebrate animals in terrestrial systems, including mammals, birds, reptiles etc.;  Aquatic Predator = species obtaining at least 60% of food resources from vertebrate and invertebrate animals in aquatic systems, including fish, crustacea, molluscs, etc;  Scavenger = species obtaining at least 60% of food resources from carrion, offal or refuse; Omnivore = Species using multiple niches, within or across trophic levels, in relatively equal proportions | - | Categorical | 0.6 |
| Mean body mass | Mean of the available data about the body mass of the scavenger species.  Composed of:  5-1_AdultBodyMass_g from Pantheria (only for mammals): Mass of adult (or age unspecified) live or freshly-killed specimens (excluding pregnant females) using captive, wild, provisioned, or unspecified populations; male, female, or sex unspecified individuals; primary, secondary, or extrapolated sources; all measures of central tendency; in all localities. [grams]  Mass from AVONET (only for birds): Body mass given as species average (incorporating both male and female body mass).  adult_body_mass_g from Amniota database: Body mass using data from males, females, and/or unspecified adults.  BodyMass.Value from Elton Traits. | grams | Continuous | 1 |
| Omnivory level | Calculated using the following variables (percentages): "Diet.Inv" , "Diet.Vend", "Diet.Vect" ,  "Diet.Vfish","Diet.Vunk", "Diet.Scav", "Diet.Fruit",  "Diet.Nect" , "Diet.Seed" , "Diet.PlantO". I assessed how many of these resource categories a species exploited (a species could exploit from 1-10 “resources”) over the total number of resource categories (10), independently of the quantity of the explotation by the species. | - | Continuous | 0.94 |
| Mean home range | Mean individual home range of adult species taken from the following sources:  Mammals – Pantheria:  22-2_HomeRange_Indiv_km2: Size of the area within which everyday activities of individuals are typically restricted, estimated by either direct observation, radio telemetry, trapping or unspecified methods over any duration of time, using non-captive populations; male, female, or sex unspecified individuals; primary, secondary, or extrapolated sources; all measures of central tendency; in all localities.  Adult home range Sebastian Gonzales (2021): Mean home range in km2 (continuous variable). They used the data from ref_1 and some other references. | Km2 | Continuous | 0.78 |
| Mean body length | Mean taken from the following databases:  Pantheria (mammals) X13.1_AdultHeadBodyLen_mm: Total length from tip of nose to anus or base of tail of adult (or age unspecified) live, freshly-killed, or museum specimens using captive, wild, provisioned, or unspecified populations; male, female, or sex unspecified individuals; primary, secondary, or extrapolated sources; all measures of central tendency; in all localities.  Amniota  Measure from tip of the snout to either the opening of the cloaca (Birds & Reptiles) or tail base (mammals) for male, female, and/or unspecified adults. | mm | Continuous | 1 |
| BMR_mLO2hr | Mammals Pantheria 18-1_BasalMetRate_mLO2hr: Basal metabolic rate of adult (or age unspecified) individual(s) using captive, wild, provisioned, or unspecified populations; male, female, or sex unspecified individuals; primary, secondary, or extrapolated sources; all measures of central tendency; in all localities. Metabolic rate was measured when individual(s) were experiencing neither heat nor cold stress (i.e. are in their thermoneutral zone); are resting and calm; and are post–absorptive (are not digesting or absorbing a meal) and data were only accepted where there was also a measure of body mass for the same individual(s). | mL(O2)/hr | Continuous | 0.7 |
| Population density | Pantheria 21-1_PopulationDensity: Number of individuals per square kilometer, estimated with either direct, indirect or unspecified counts, measured in any area size within a human, ecological or unspecified boundary, over any duration of time, using non-captive, non-provisioned populations; male, female, or sex unspecified individuals; primary, secondary, or extrapolated sources; all measures of central tendency; in all localities. | n/km2 | Continuous | 0.2 |
| extremetiesbody_ratio | Length of extremities (variable “extremeties”) over the length of the body (variable “mean_leangth”). | - | Continuous | 0.89 |
| Extremeties_tail_ratio | Length of extremities (variable “extremeties”) over the length of the tail (variable “Tail.Length”). | - | Continuous | 0.8 |
| Trophic.Level | Trophic level of each species measured using any qualitative or quantitative dietary measure. Species were defined as “herbivore “(not vertebrate and/or invertebrate), “omnivore “(vertebrate and/or invertebrate plus any of the other categories) and “carnivore” (vertebrate and/or invertebrate only). |  | Categorical | 1 |
| Extremities | Extremeties’ length. For birds, it refers to the wing length as defined in the database Avonet (see below), and for mammals, to the limb length defined as the shoulder height.  Wing length: Length from the carpal joint (bend of the wing) to the tip of the longest primary on the unflattened wing [mm]. | mm | Continuous | 0.88 |
| Conservation status | The conservation status of species as defined by the Spanish Ministry for the Ecological Transition and the demographic Challenge (MITECO).  "3" : Endangered  "2”: Vulnerable  "1" : Listed in LESPRE  "0”: Not particular protection | - | Categorical | 1 |

##
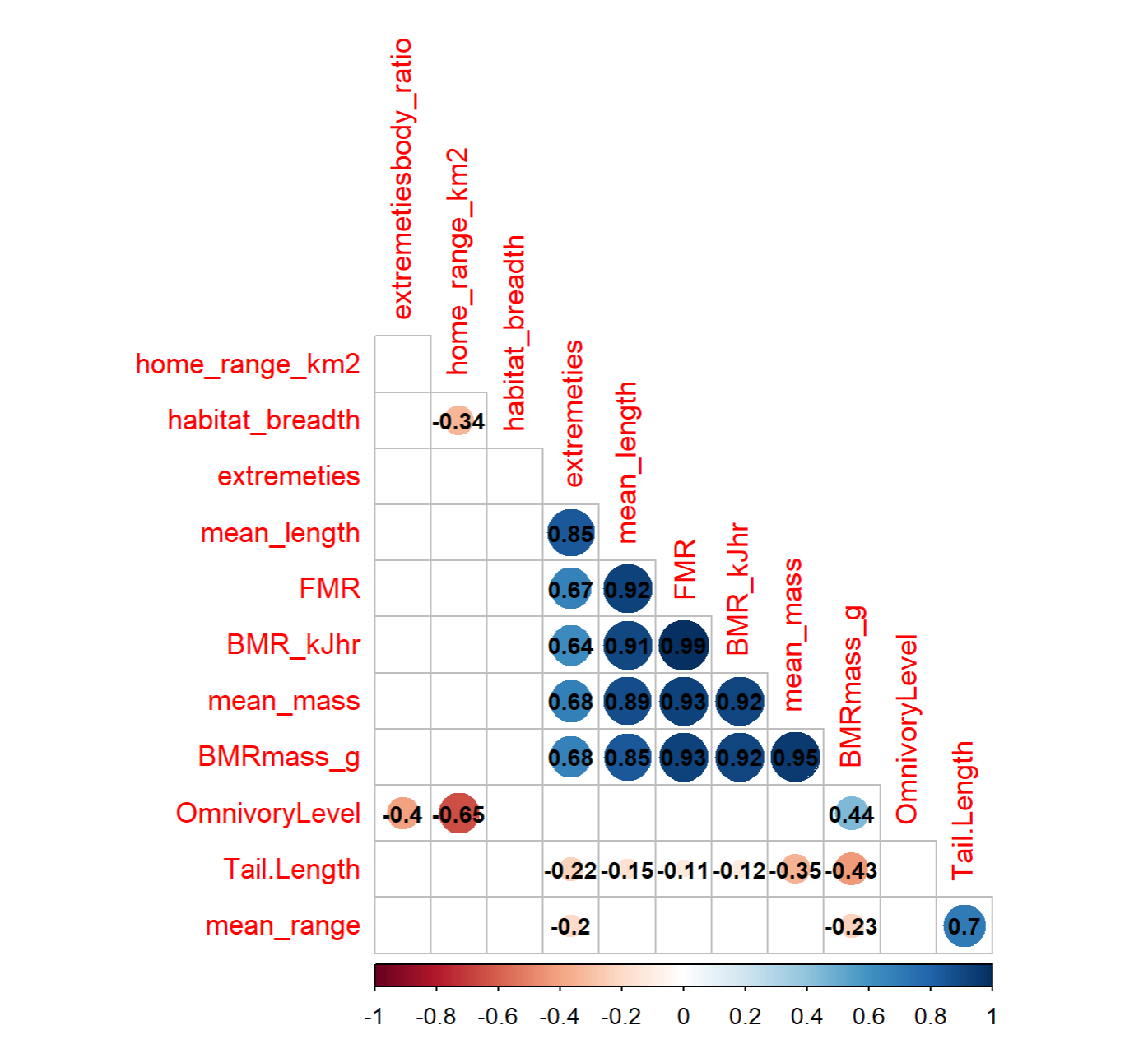


**Figure S1.** Corrplot of the functional traits with completeness >70%. Displayed are only the coefficients of the correlations that were significant (p-value < 0.5).

**Table S3.** Models AICs used for selection of the random component. We selected the two models in the first two rows. *class was included in the fixed effects only when it was not in the random effects.

| **Fixed effects** | **Random intercept** | **Random slope** | **AIC** |
| --- | --- | --- | --- |
| omnivory level + log(mean mass) + extremities-body ratio +  log(home range)+ habitat breadth + activity + social +  predator +  class+*  sight +  noise +  status | network | - | 86.76556 |
|  | class | - | 86.52263 |
|  | network and class | - | 87.14865 |
|  | class | omnivory level | 90.52263 |
|  | class | mean mass | 90.52263 |
|  | class | extremities-body ratio | 90.52263 |
|  | class | home range | 90.52263 |
|  | class | habitat breadth | 90.52263 |
|  | network and class | omnivory level | 91.14865 |
|  | network and class | mean mass | 91.14865 |
|  | network and class | extremeties-body ratio | 91.14865 |
|  | network and class | home range | 91.14865 |
|  | network and class | Habitat breadth | 91.14865 |

**Table S4.** Formulas used in function glmulti for model averaging. In formulas 2 and 3, ‘class’ represents smell and mobility (all mammals had good olfaction and were terrestrial).

| **Formula** | **Variables** |
| --- | --- |
| 1 | PC1 ~ OmnivoryLevel + log(mean_mass) + extremetiesbody_ratio + log(home_range_km2) + (habitat_breadth) + activity +social + predator+ class+ sight +noise +status + (1\|network) |
| 2 | PC1 ~ OmnivoryLevel + log(mean_mass) + extremetiesbody_ratio + log(home_range_km2) + (habitat_breadth) + activity +social + predator+ sight +noise +status + (1\|class) |
| 3 | PC1 ~ OmnivoryLevel + log(mean_mass) + extremetiesbody_ratio + log(home_range_km2) + (habitat_breadth) + activity +social + predator+ sight +noise +status + (1\|class) + (1\|network) |

**Table S5.** Top two models within 2 IC produced by *glmulti* using the first formula in Table S4 with ‘network’ as a random component.


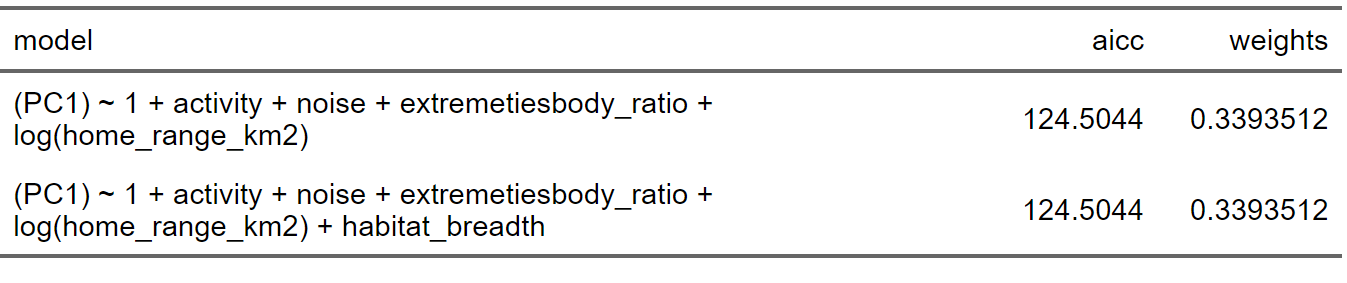


**Table S6.** Averaged parameter estimates using the top two models displayed in Table S5.


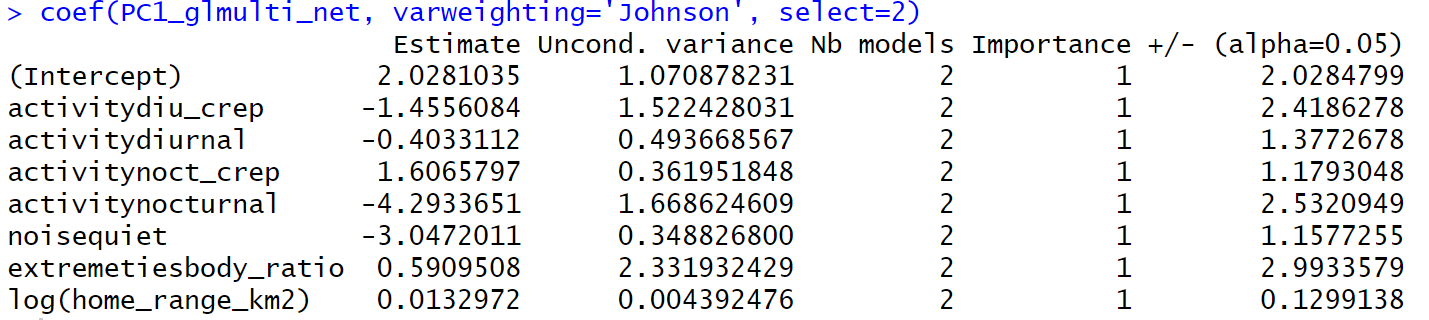


**Table S7.** Top two models within 2 IC produced by *glmulti* using the second formula in Table S4 with ‘class’ as a random component.


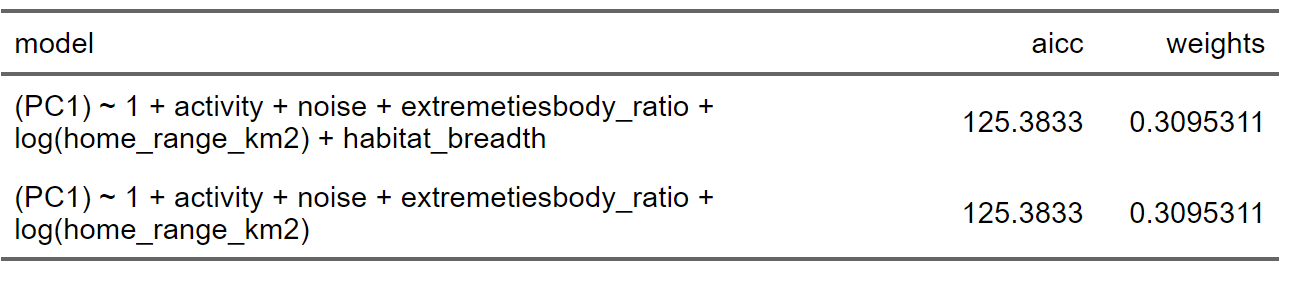


**Table S8.** Averaged parameter estimates of the top two models of Table S7.


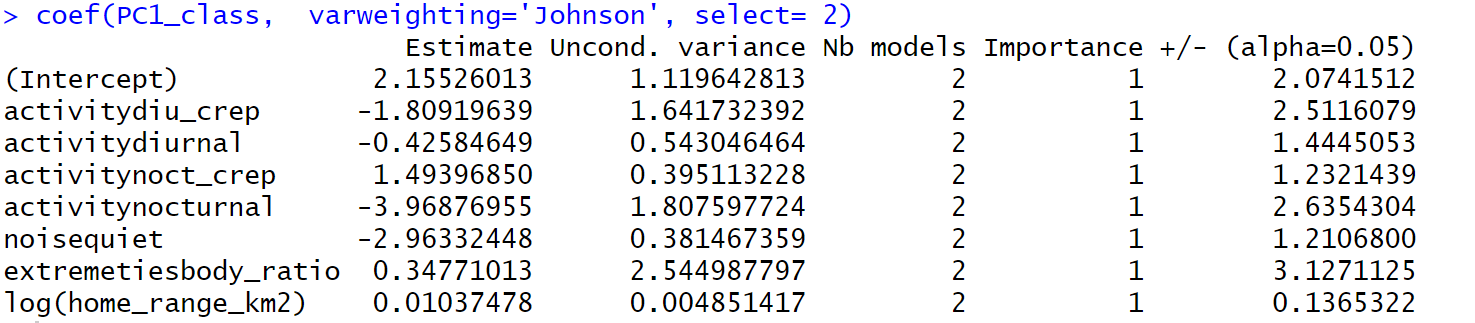


**Table S9.** Top two models within 2 IC produced by *glmulti* using the third formula in Table S4 with ‘class’ and ‘network’ as random components. This formula was used for the final analysis.


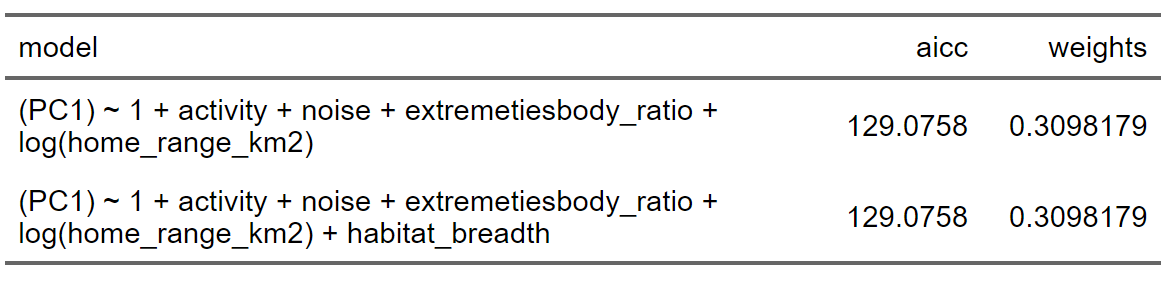


**Table S10.** Averaged parameter estimates of the top two models displayed in Table S9.


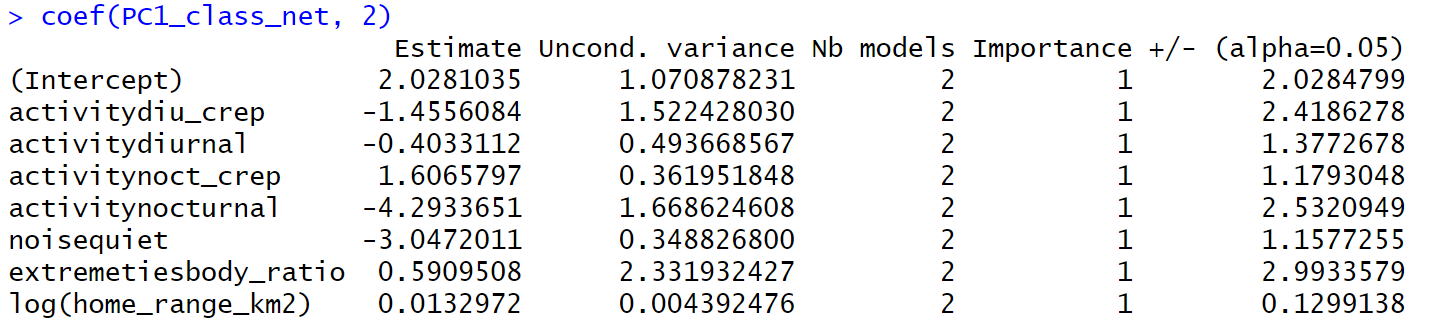


**Table S11.** Summary of the continuous explanatory variables used in the analysis.

| **Trait** | **Min** | **Max** | **Mean** | **Median** | **SD** |
| --- | --- | --- | --- | --- | --- |
| Home range [km^2^] | 0.011 | 46735.76 | 4501.02 | 9.32 | 12,192.32 |
| Body mass [kg] | 0.022 | 205.8 | 13.58 | 2.013 | 35.922 |
| Extremities-body ratio | 0.25 | 0.84 | 0.63 | 0.66 | 0.163 |
| Omnivory level | 0.1 | 0.8 | 0.3529 | 0.4 | - |

**Table S12.** Number of individuals displaying the different categories of the categorical explanatory variables used in the analysis.

| **Trait** | **n** |
| --- | --- |
| Olfaction - low | 33 |
| Olfaction - high | 21 |
| Foraging behaviour - social | 23 |
| Foraging behaviour - solitary | 31 |
| Non-predator | 10 |
| Meso-predator | 35 |
| Apex-predator | 9 |
| Activity - diurnal | 33 |
| Activity – diurnal and crepuscular | 1 |
| Activity – cathemeral | 4 |
| Activity – nocturnal | 6 |
| Activity - nocturnal and crepuscular | 10 |
| Sight – high | 45 |
| Sight – low | 6 |
| Noisiness – noisy | 15 |
| Noisiness – quiet | 35 |
| Conservation status – none | 30 |
| Conservation status – LESPRE | 14 |
| Conservation status – Vulnerable | 5 |
| Conservation status – Endangered | 5 |

**Table S13.** Species-level network metrics d', normalized degree, closeness and betweenness for the 54 individuals in all three networks. In species ID, CC stands for Cordillera Cantábrica, MT for Montes de Toledo and SM for Sierra Morena.

| **Species ID** | **d’** | **ND** | **Closeness** | **Betweenness** |
| --- | --- | --- | --- | --- |
| Accipitergentilis_MT | 0 | 1 | 0.0078123 | 0.066666667 |
| Aegypiusmonachus_MT | 0.002267 | 1 | 0.3183817 | 0.066666667 |
| Aegypiusmonachus_SM | 0.051702 | 1 | 0.1062601 | 0.083333333 |
| Aquilaadalberti_MT | 0.078279 | 1 | 0.0194404 | 0.066666667 |
| Aquilachrysaetos_SM | 0 | 1 | 0.0256324 | 0.083333333 |
| Buteobuteo_CC | 0.110791 | 1 | 0.108433 | 0.097902098 |
| Buteobuteo_MT | 0.032143 | 1 | 0.0117003 | 0.066666667 |
| Canisfamiliaris_SM | 0.006514 | 1 | 0.0340224 | 0.083333333 |
| Circusaeruginosus_MT | 0 | 1 | 0.0078123 | 0.066666667 |
| Corvuscorax_CC | 0.052098 | 1 | 0.4375113 | 0.097902098 |
| Corvuscorax_MT | 0.010049 | 1 | 0.0906575 | 0.066666667 |
| Corvuscorone_CC | 0.054279 | 1 | 0.3763867 | 0.097902098 |
| Cyanopicacyanus_MT | 0.030628 | 1 | 0.0499291 | 0.066666667 |
| Cyanopicacyanus_SM | 0.000303 | 1 | 0.4074441 | 0.083333333 |
| Garrulusglandarius_MT | 0.007524 | 1 | 0.0309614 | 0.066666667 |
| Garrulusglandarius_SM | 0.03944 | 1 | 0.0587444 | 0.083333333 |
| Genettagenetta_MT | 0 | 1 | 0.0078123 | 0.066666667 |
| Genettagenetta_SM | 0.018399 | 1 | 0.042337 | 0.083333333 |
| Gypsfulvus_CC | 0.155479 | 1 | 1 | 0.097902098 |
| Gypsfulvus_MT | 0.009951 | 1 | 1 | 0.066666667 |
| Gypsfulvus_SM | 0.000172 | 1 | 1 | 0.083333333 |
| Herpestesichneumon_SM | 0 | 1 | 0.0256324 | 0.083333333 |
| Martesfoina_CC | 0.093366 | 1 | 0.0893972 | 0.097902098 |
| Martesfoina_MT | 0 | 1 | 0.0117003 | 0.066666667 |
| Martesfoina_SM | 0.004966 | 1 | 0.0668391 | 0.083333333 |
| Milvusmigrans_MT | 0.036927 | 1 | 0.0232926 | 0.066666667 |
| Picapica_MT | 0.010049 | 1 | 0.0906575 | 0.066666667 |
| Picapica_SM | 0.042656 | 1 | 0.1062601 | 0.083333333 |
| Susscrofa_MT | 0.03696 | 1 | 0.1854123 | 0.066666667 |
| Susscrofa_SM | 0.000396 | 1 | 0.3522175 | 0.083333333 |
| Vulpesvulpes_CC | 0.12107 | 1 | 0.3300043 | 0.097902098 |
| Vulpesvulpes_MT | 0.012736 | 1 | 0.2313203 | 0.066666667 |
| Vulpesvulpes_SM | 0.009485 | 1 | 0.3121478 | 0.083333333 |
| Aquilachrysaetos_CC | 0.041828 | 0.75 | 0.0491842 | 0.020979021 |
| Canisfamiliaris_CC | 0.080558 | 0.75 | 0.0700048 | 0.097902098 |
| Canislupus_CC | 0.029841 | 0.75 | 0.0649004 | 0.097902098 |
| Picapica_CC | 0.031882 | 0.75 | 0.1279851 | 0.097902098 |
| Susscrofa_CC | 0.075192 | 0.75 | 0.1763269 | 0.097902098 |
| Apodemusspp_SM | 0.074863 | 0.5 | 0.0086456 | 0 |
| Aquilaadalberti_SM | 0.074863 | 0.5 | 0.0086456 | 0 |
| Aquilachrysaetos_MT | 0 | 0.5 | 0.0039142 | 0 |
| Aquilafasciata_SM | 0 | 0.5 | 0.0086384 | 0 |
| Bubobubo_SM | 0 | 0.5 | 0.0086384 | 0 |
| Canisfamiliaris_MT | 0.08621 | 0.5 | 0.0039164 | 0 |
| Corvuscorax_SM | 0.171843 | 0.5 | 0.0172589 | 0 |
| Feliscatus_SM | 0 | 0.5 | 0.0086384 | 0 |
| Milvusmilvus_MT | 0 | 0.5 | 0.0039142 | 0 |
| Neophronpercnopterus_MT | 0.08621 | 0.5 | 0.0039164 | 0 |
| Aegypiusmonachus_CC | 0 | 0.25 | 0.0056827 | 0 |
| Apodemusspp_CC | 0 | 0.25 | 0.0056827 | 0 |
| Genettagenetta_CC | 0.193198 | 0.25 | 0.0056999 | 0 |
| Milvusmilvus_CC | 0.193198 | 0.25 | 0.0056999 | 0 |
| Neophronpercnopterus_CC | 0.149711 | 0.25 | 0.0056829 | 0 |
| Ursusarctos_CC | 0.03652 | 0.25 | 0.0113197 | 0 |

**Table S14.** PCA loadings of the four metrics used for the analysis.

| **Metric** | **PC1** | **PC2** | **PC3** | **PC4** |
| --- | --- | --- | --- | --- |
| d’ | -0.26178 | 0.826507 | 0.474961 | -0.15089 |
| normalized.degree | 0.63046 | -0.09139 | 0.278132 | -0.7189 |
| closeness | 0.394095 | 0.547324 | -0.73826 | -0.00959 |
| betweenness | 0.615369 | 0.094712 | 0.3899 | 0.678475 |

**Table S15.** Abbreviations used for the PCA biplot (Figure 4, Results).

| Species’ name | Abbreviation |
| --- | --- |
| *Accipiter gentilis* | *A. gentilis* |
| *Aegypius monachus* | *A. monachus* |
| *Apodemus spp.* | *A. spp.* |
| *Aquila adalberti* | *A. adalberti* |
| *Aquila chrysaetos* | *A. chrysaetos* |
| *Aquila fasciata* | *A. fasciata* |
| *Bubo bubo* | *B. bubo* |
| *Buteo buteo* | *B. buteo* |
| *Canis familiaris* | *C. familiaris* |
| *Canis lupus* | *C. lupus* |
| *Circus aeruginosus* | *C. aeruginosus* |
| *Corvus corax* | *C. corax* |
| *Corvus corone* | *C. corone* |
| *Cyanopica cyanus* | *C. cyanus* |
| *Felis catus* | *F. catus* |
| *Garrulus glandarius* | *G. glandarius* |
| *Genetta genetta* | *G. genetta* |
| *Gyps fulvus* | *G. fulvus* |
| *Herpestes ichneumon* | *H. ichneumon* |
| *Martes foina* | *M. foina* |
| *Milvus migrans* | *M. migrans* |
| *Milvus milvus* | *M. milvus* |
| *Neophron percnopterus* | *N. percnopterus* |
| *Pica pica* | *P. pica* |
| *Sus scrofa* | *S. scrofa* |
| *Ursus arctos* | *U. arctos* |
| *Vulpes vulpes* | *V. vulpes* |

**Table S16.** PCA scores for each individual observed in the networks. Red indicates high values, blue indicates low values. High values stand for centrality, while low values for peripherality.

| **Species_ID** | **PC1** | **PC2** | **PC3** | **PC4** |
| --- | --- | --- | --- | --- |
| Accipitergentilis_MT | 0.628813 | -1.01485 | 0.32807 | -0.19467 |
| Aegypiusmonachus_CC | -2.14416 | -0.93455 | -1.0909 | 0.589506 |
| Aegypiusmonachus_MT | 1.124309 | -0.27607 | -0.60081 | -0.21341 |
| Aegypiusmonachus_SM | 0.800046 | 0.050779 | 0.655674 | -0.05322 |
| Apodemusspp_CC | -2.14416 | -0.93455 | -1.0909 | 0.589506 |
| Apodemusspp_SM | -1.93673 | 0.150551 | -0.18112 | -0.27294 |
| Aquilaadalberti_MT | 0.263253 | 0.225542 | 0.990198 | -0.41677 |
| Aquilaadalberti_SM | -1.93673 | 0.150551 | -0.18112 | -0.27294 |
| Aquilachrysaetos_CC | -0.80489 | -0.30137 | -0.13678 | -0.46414 |
| Aquilachrysaetos_MT | -1.57669 | -1.02123 | -0.83388 | -0.06078 |
| Aquilachrysaetos_SM | 0.922494 | -0.93375 | 0.441271 | 0.096367 |
| Aquilafasciata_SM | -1.56899 | -1.01053 | -0.84832 | -0.06097 |
| Bubobubo_SM | -1.56899 | -1.01053 | -0.84832 | -0.06097 |
| Buteobuteo_CC | 0.744632 | 1.007722 | 1.322218 | 0.034412 |
| Buteobuteo_MT | 0.477262 | -0.50753 | 0.60266 | -0.28583 |
| Canisfamiliaris_CC | 0.2601 | 0.534455 | 0.918591 | 0.771896 |
| Canisfamiliaris_MT | -2.00018 | 0.315824 | -0.06553 | -0.30488 |
| Canisfamiliaris_SM | 0.90418 | -0.81371 | 0.47369 | 0.07759 |
| Canislupus_CC | 0.500915 | -0.2637 | 0.482164 | 0.915701 |
| Circusaeruginosus_MT | 0.628813 | -1.01485 | 0.32807 | -0.19467 |
| Corvuscorax_CC | 1.569772 | 0.842998 | -0.20652 | 0.187536 |
| Corvuscorax_MT | 0.714595 | -0.6713 | 0.164463 | -0.22641 |
| Corvuscorax_SM | -2.39907 | 1.674149 | 0.656896 | -0.54787 |
| Corvuscorone_CC | 1.459349 | 0.738332 | -0.0003 | 0.183788 |
| Cyanopicacyanus_MT | 0.547062 | -0.4444 | 0.472342 | -0.28306 |
| Cyanopicacyanus_SM | 1.543857 | -0.06403 | -0.72282 | 0.08036 |
| Feliscatus_SM | -1.56899 | -1.01053 | -0.84832 | -0.06097 |
| Garrulusglandarius_MT | 0.629615 | -0.8457 | 0.324388 | -0.21689 |
| Garrulusglandarius_SM | 0.782768 | -0.24704 | 0.691593 | -0.01662 |
| Genettagenetta_CC | -3.09318 | 2.061841 | 0.630935 | 0.042484 |
| Genettagenetta_MT | 0.628813 | -1.01485 | 0.32807 | -0.19467 |
| Genettagenetta_SM | 0.859364 | -0.61055 | 0.554201 | 0.043611 |
| Gypsfulvus_CC | 1.979525 | 3.720704 | -1.00407 | -0.12749 |
| Gypsfulvus_MT | 2.198488 | 1.387355 | -2.6153 | -0.26222 |
| Gypsfulvus_SM | 2.511133 | 1.276425 | -2.53479 | 0.057217 |
| Herpestesichneumon_SM | 0.922494 | -0.93375 | 0.441271 | 0.096367 |
| Martesfoina_CC | 0.799173 | 0.694356 | 1.225095 | 0.084503 |
| Martesfoina_MT | 0.635156 | -1.00604 | 0.316188 | -0.19483 |
| Martesfoina_SM | 0.965321 | -0.76337 | 0.359604 | 0.080672 |
| Milvusmigrans_MT | 0.47267 | -0.40707 | 0.609876 | -0.29984 |
| Milvusmilvus_CC | -3.09318 | 2.061841 | 0.630935 | 0.042484 |
| Milvusmilvus_MT | -1.57669 | -1.02123 | -0.83388 | -0.06078 |
| Neophronpercnopterus_CC | -2.87959 | 1.387345 | 0.243403 | 0.165615 |
| Neophronpercnopterus_MT | -2.00018 | 0.315824 | -0.06553 | -0.30488 |
| Picapica_CC | 0.593795 | -0.08911 | 0.307579 | 0.907416 |
| Picapica_MT | 0.714595 | -0.6713 | 0.164463 | -0.22641 |
| Picapica_SM | 0.844484 | -0.08952 | 0.575049 | -0.02761 |
| Susscrofa_CC | 0.459904 | 0.692109 | 0.54585 | 0.782871 |
| Susscrofa_MT | 0.736974 | -0.03926 | 0.114743 | -0.30637 |
| Susscrofa_SM | 1.453306 | -0.1877 | -0.55322 | 0.082287 |
| Ursusarctos_CC | -2.31436 | -0.35538 | -0.78264 | 0.485879 |
| Vulpesvulpes_CC | 1.055584 | 1.669136 | 0.736729 | -0.00349 |
| Vulpesvulpes_MT | 0.930857 | -0.31094 | -0.24144 | -0.2396 |
| Vulpesvulpes_SM | 1.343294 | -0.13752 | -0.34977 | 0.058143 |

**Table S17.** Tests used to search for differences in centrality among different groups. We tested for differences among classes, scavengers (obligate and facultative) and species with different conservation status For the first two we used a Wilcoxon test, for the third a Kruskal-Wallis test. A Shapiro -test was used to assess the normality of PC1. The significance of the test signalled the non-normality of the data. Thus, non-parametric tests were used.

| **Test** | | **Value** |
| --- | --- | --- |
| Shapiro Test for PC1 | Shapiro test’s W | 0.86952 |
|  | p-value | 2.947e-05 |
| Wilcoxon’s test for PC1~ class | Wilcoxon test’s W | 342.5 |
|  | Wilcoxon test’s p-value | 0.9505 |
| Wilcoxon’s test for PC1~scavenging | Wilcoxon test’s W | 144 |
|  | Wilcoxon test’s p-value | 0.336 |
| Kruskal-Wallis test for PC1~status | Chi-squared | 9.1949 |
|  | p-value | 0.02681 |
|  | df | 3 |

**Table S18.** Correlation coefficients between the centrality metrics normalized degree (ND), specialisation D', closeness C_c_ and betweenness C_B_ centrality (green cells), and their respective p-values (blue cells). In bold are the significant correlations.

| **Metric** | **ND** | **D’** | **C_c_** | **C_B_** |
| --- | --- | --- | --- | --- |
| ND | 1 | 0.009651 | 0.005576 | 2.2e-16 |
| D’ | **-0.3492038** | 1 | 0.9314 | 0.2137 |
| C_c_ | **0.3722372** | 0.01199322 | 1 | 0.002097 |
| C_B_ | **0.8703354** | -0.1719678 | 0.4096516 | 1 |

##

### References

Allesina, S., & Tang, S. (2012). Stability criteria for complex ecosystems. *Nature*, *483*(7388), 205–208.

Billerman, M., Keeney, B. K., Rodewald, P. G., & Schulenberg, T. S. (2022). *Birds of the World. Cornell Lab of Ornithology, Ithaca*.

Blüthgen, N., Menzel, F., & Blüthgen, N. (2006). Measuring specialization in species interaction networks. *BMC Ecology*, *6*. https://doi.org/10.1186/1472-6785-6-9

Boletín Oficial Del Estado. (2011). Real Decreto 139/2011, de 4 de febrero, para el desarrollo del Listado de Especies Silvestres en Régimen de Protección Especial y del Catálogo Español de Especies Amenazadas. *BOE*, *46*, 20912–20951.

Calcagno, V., & de Mazancourt, C. (2010). glmulti: An R Package for Easy Automated Model Selection with (Generalized) Linear Models. *Journal of Statistical Software*, *34*(12), 1–29. https://doi.org/10.18637/jss.v034.i12

Cirtwill, A. R., Dalla Riva, G. V., Gaiarsa, M. P., Bimler, M. D., Cagua, E. F., Coux, C., & Dehling, D. M. (2018). A review of species role concepts in food webs. *Food Webs*, *16*, e00093. https://doi.org/https://doi.org/10.1016/j.fooweb.2018.e00093

Costa, L. D. F., Rodrigues, F. A., Travieso, G., & Boas, P. R. V. (2007). Characterization of complex networks: A survey of measurements. *Advances in Physics*, *56*(1), 167–242. https://doi.org/10.1080/00018730601170527

Dormann, C. F., Fründ, J., Blüthgen, N., & Gruber, B. (2009). Indices, Graphs and Null Models: Analyzing Bipartite Ecological Networks. *The Open Journal of Ecology*, *2*, 7–24. https://doi.org/http://dx.doi.org/10.2174/1874213000902010007

Estrada, E., & Bodin, Ö. (2008). Using network centrality measures to manage landscape connectivity. *Ecological Applications*, *18*(7), 1810–1825.

Gaston, K. J., & Blackburn, T. M. (1997). Birds, body size and the threat of extinction. *Philosophical Transactions of the Royal Society of London. Series B: Biological Sciences*, *347*(1320), 205–212. https://doi.org/10.1098/rstb.1995.0022

IUCN Standards and Petitions Committee. (2022). *Guidelines for Using the IUCN Red List Categories and Criteria*.

Jones, K. E., Bielby, J., Cardillo, M., Fritz, S. A., O’Dell, J., Orme, C. D. L., Safi, K., Sechrest, W., Boakes, E. H., & Carbone, C. (2009). PanTHERIA: a species‐level database of life history, ecology, and geography of extant and recently extinct mammals: Ecological Archives E090‐184. *Ecology*, *90*(9), 2648.

Marti, R., & del Moral, J. C. (2003). *Atlas de las aves reproductoras de Espana*.

Medeiros, L. P., Garcia, G., Thompson, J. N., & Guimaraes Jr, P. R. (2018). The geographic mosaic of coevolution in mutualistic networks. *Proceedings of the National Academy of Sciences*, *115*(47), 12017–12022.

Moulatlet, G. M., Dáttilo, W., & Villalobos, F. (2023). Species‐level drivers of avian centrality within seed‐dispersal networks across different levels of organisation. *Journal of Animal Ecology*, *92*(11), 2126–2137.

Myers, P., Espinosa, R., Parr, C. S., Jones, T., Hammond, G. S., & Dewey, T. A. (2006). The animal diversity web. *Accessed October*, *12*(2006), 2.

Myhrvold, N. P., Baldridge, E., Chan, B., Sivam, D., Freeman, D. L., & Ernest, S. K. M. (2015). An amniote life‐history database to perform comparative analyses with birds, mammals, and reptiles: Ecological Archives E096‐269. *Ecology*, *96*(11), 3109.

RStudio Team. (2022). *RStudio: Integrated Development for R. RStudio, PBC, Boston, MA* . http://www.rstudio.com/

Sebastián-González, E., Morales-Reyes, Z., Botella, F., Naves-Alegre, L., Pérez-García, J. M., Mateo-Tomás, P., Olea, P. P., Moleón, M., Barbosa, J. M., Hiraldo, F., Arrondo, E., Donázar, J. A., Cortés-Avizanda, A., Selva, N., Lambertucci, S. A., Bhattacharjee, A., Brewer, A. L., Abernethy, E. F., Turner, K. L., … Sánchez-Zapata, J. A. (2021). Functional traits driving species role in the structure of terrestrial vertebrate scavenger networks. *Ecology*, *102*(12), 1–12. https://doi.org/10.1002/ecy.3519

Tobias, J. A., Sheard, C., Pigot, A. L., Devenish, A. J. M., Yang, J., Sayol, F., Neate-Clegg, M. H. C., Alioravainen, N., Weeks, T. L., Barber, R. A., Walkden, P. A., MacGregor, H. E. A., Jones, S. E. I., Vincent, C., Phillips, A. G., Marples, N. M., Montaño-Centellas, F. A., Leandro-Silva, V., Claramunt, S., … Schleuning, M. (2022). AVONET: morphological, ecological and geographical data for all birds. *Ecology Letters*, *25*(3), 581–597. https://doi.org/https://doi.org/10.1111/ele.13898

Wilman, H., Belmaker, J., Simpson, J., de la Rosa, C., Rivadeneira, M. M., & Jetz, W. (2014). EltonTraits 1.0: Species‐level foraging attributes of the world’s birds and mammals: Ecological Archives E095‐178. *Ecology*, *95*(7), 2027.
